# Substitution of PINK1 Gly411 modulates substrate receptivity and turnover

**DOI:** 10.1080/15548627.2022.2151294

**Published:** 2022-12-05

**Authors:** Fabienne C Fiesel, Dominika Fričová, Caleb S Hayes, Mathew A Coban, Roman Hudec, Jenny M Bredenberg, Benjamin J Broadway, Briana N Markham, Tingxiang Yan, Paige K Boneski, Gabriella Fiorino, Jens O Watzlawik, Xu Hou, Arthur M McCarty, Laura J Lewis-Tuffin, Jun Zhong, Benjamin J Madden, Alban Ordureau, Heeseon An, Andreas Puschmann, Zbigniew K Wszolek, Owen A Ross, J Wade Harper, Thomas R Caulfield, Wolfdieter Springer

**Affiliations:** a Department of Neuroscience, Mayo Clinic, Jacksonville, FL, USA; b Neuroscience PhD Program, Mayo Graduate School of Biomedical Sciences, Mayo Clinic, Jacksonville, FL, USA; c Department of Cancer Biology, Mayo Clinic, Jacksonville, FL, USA; d Cytometry and Imaging Laboratory, Department of Research, Mayo Clinic, Jacksonville, FL, USA; e Department of Laboratory Medicine and Pathology, Mayo Clinic, Rochester, MN, USA; f Proteomics Core, Medical Genome Facility, Mayo Clinic, Rochester, MN, USA; g Department of Cell Biology, Harvard Medical School, Boston, MA, USA; h Department of Neurology, Lund University, Skane University Hospital, Sweden; i Department of Neurology, Mayo Clinic, Jacksonville, FL, USA; j Department of Neurosurgery, Mayo Clinic, Jacksonville, FL, USA

**Keywords:** Mitophagy, parkin, parkinson disease, PINK1, PRKN, ubiquitin

## Abstract

The ubiquitin (Ub) kinase-ligase pair PINK1-PRKN mediates the degradation of damaged mitochondria by macroautophagy/autophagy (mitophagy). PINK1 surveils mitochondria and upon stress accumulates on the mitochondrial surface where it phosphorylates serine 65 of Ub to activate PRKN and to drive mitochondrial turnover. While loss of either PINK1 or PRKN is genetically linked to Parkinson disease (PD) and activating the pathway seems to have great therapeutic potential, there is no formal proof that stimulation of mitophagy is always beneficial. Here we used biochemical and cell biological methods to study single nucleotide variants in the activation loop of PINK1 to modulate the enzymatic function of this kinase. Structural modeling and *in vitro* kinase assays were used to investigate the molecular mechanism of the PINK1 variants. In contrast to the PD-linked PINK1^G411S^ mutation that diminishes Ub kinase activity, we found that the PINK1^G411A^ variant significantly boosted Ub phosphorylation beyond levels of PINK1 wild type. This resulted in augmented PRKN activation, mitophagy rates and increased viability after mitochondrial stress in midbrain-derived, gene-edited neurons. Mechanistically, the G411A variant stabilizes the kinase fold of PINK1 and transforms Ub to adopt the preferred, C-terminally retracted conformation for improved substrate turnover. In summary, we identify a critical role of residue 411 for substrate receptivity that may now be exploited for drug discovery to increase the enzymatic function of PINK1. The genetic substitution of Gly411 to Ala increases mitophagy and may be useful to confirm neuroprotection *in vivo* and might serve as a critical positive control during therapeutic development.

**Abbreviations**: ATP: adenosine triphosphate; CCCP: carbonyl cyanide m-chlorophenyl hydrazone; Ub-CR: ubiquitin with C-terminally retracted tail; CTD: C-terminal domain (of PINK1); ELISA: enzyme-linked immunosorbent assay; HCI: high-content imaging; IB: immunoblot; IF: immunofluorescence; NPC: neuronal precursor cells; MDS: molecular dynamics simulation; PD: Parkinson disease; p-S65-Ub: ubiquitin phosphorylated at Ser65; RMSF: root mean scare fluctuation; TOMM: translocase of outer mitochondrial membrane; TVLN: ubiquitin with T66V and L67N mutation, mimics Ub-CR; Ub: ubiquitin; WT: wild-type.

## Introduction

PINK1-PRKN mitophagy is a stress-activated pathway that identifies and selectively labels damaged mitochondria for their safe disposal through the autophagy-lysosome system (reviewed in [1]). Failure of this critical quality control mechanism is thought to lead to the buildup of macromolecular damage over time, eventually culminating in cell death. As such, complete loss of either enzyme, PINK1 or PRKN, is invariably linked to early-onset Parkinson disease (PD). Although dopamine neurons appear exquisitely sensitive to loss of mitophagy, emerging evidence suggest a much broader contribution to various age-related diseases of the brain and beyond. In line with this notion, PINK1 and PRKN are widely expressed and their reduction in cell and animal models provides a sensitized background to a variety of stressors, while overexpression of particularly PRKN, but also PINK1 [1,2], seems broadly cytoprotective. However, both enzymes are tightly regulated, and it remains uncertain whether their continuous overactivation and the potentially resulting excessive mitophagy is beneficial at all times or can be detrimental under certain circumstances.

The kinase PINK1 acts as a sensor and is continuously imported into healthy mitochondria where it is cleaved, released back to the cytosol, and rapidly degraded. Stress and damage prevent this initial import leaving full-length PINK1 to swiftly accumulate locally in the outer mitochondrial membrane with its kinase domain facing the cytosol. For activation, PINK1 dimerizes into a higher molecular weight complex and autophosphorylates [3–6] to enable its kinase activity toward ubiquitin (Ub) [7–9]. Ub phosphorylated at Ser65 (p-S65-Ub) acts as an allosteric activator of and receptor for the E3 Ub ligase PRKN [10–12] providing a highly selective label [13–15]. Once recruited to damaged mitochondria, PRKN is also phosphorylated by PINK1 in its Ub-like domain [16–19]. Both phosphorylation events are required to activate PRKN from its cytosolic, auto-inhibited state and to fully unleash its E3 Ub ligase functions on mitochondria. The functions of both enzymes then synergize in a feedforward loop where the products of PRKN activity become the substrates for PINK1, coating damaged mitochondria with p-S65-Ub that then also serves as a mitophagy tag [14,15,20].

Although the human structure has remained elusive, crystal structures of insect orthologs provided first insights into the kinase activity of PINK1 [21–23] and recent studies detailed the initial activation process as the first step in the mitophagy cascade [7–9]. Despite sequence differences, it has become clear that PINK1 is quite distinctive from other known kinases (reviewed in [24]). Human PINK1 contains three unique insertions in the N-lobe of its kinase domain that seem to play major roles in governing its enzymatic function. Insertion loop 2 is required for PINK1 dimerization which facilitates autophosphorylation *in trans* within its catalytic loop (S228), and not in the activation loop as the majority of kinases [25]. The resulting conformational changes lead to organization of insertion loop 3 and likely to dissociation of the dimer for activated PINK1 to engage with Ub. Moreover, it has been suggested that the preferred substrate of PINK1 is a minor conformation of Ub (less than 1%) with a C-terminal retracted tail that results in greater exposure and accessibility of the S65 residue [26]. While the PINK1-dependent phosphorylation may also change structure and function of Ub, such a conformational constraint could be rate-limiting, despite the vast abundance of Ub in cells.

Recently we had identified a partial dominant negative effect exerted by a heterozygous PINK1 activation loop mutation [27]. In probing the pathogenic mechanism further, we now report that PINK1^G411S^ is aberrantly autophosphorylated, which leads to reduced substrate phosphorylation. More importantly, we discovered that PINK1^G411A^ exhibits strikingly enhanced Ub kinase activity compared to the wild-type (WT) form. This effect was conserved across different cell types and translated into enhanced PRKN activation and increased mitophagy rates under endogenous conditions. Structural computational analyses suggested the induction of a conformational change in Ub when bound to PINK1^G411A^ resulting in increased receptivity and overall better positioning of the substrate S65. Kinase assays confirmed that in contrast to PINK1 WT, the enzymatic activity was not limited to the C-terminally retracted conformation of Ub, but instead PINK1^G411A^ was able to phosphorylate the common, extended form of Ub comparably well. Our work provides a genetic proof of concept for enhanced PINK1 activity under endogenous conditions that can potentially guide the development of small molecules changing receptivity and ultimately availability of substrates.

## Results

### PINK1^G411S^ is aberrantly autophosphorylated

**Figure 1.**
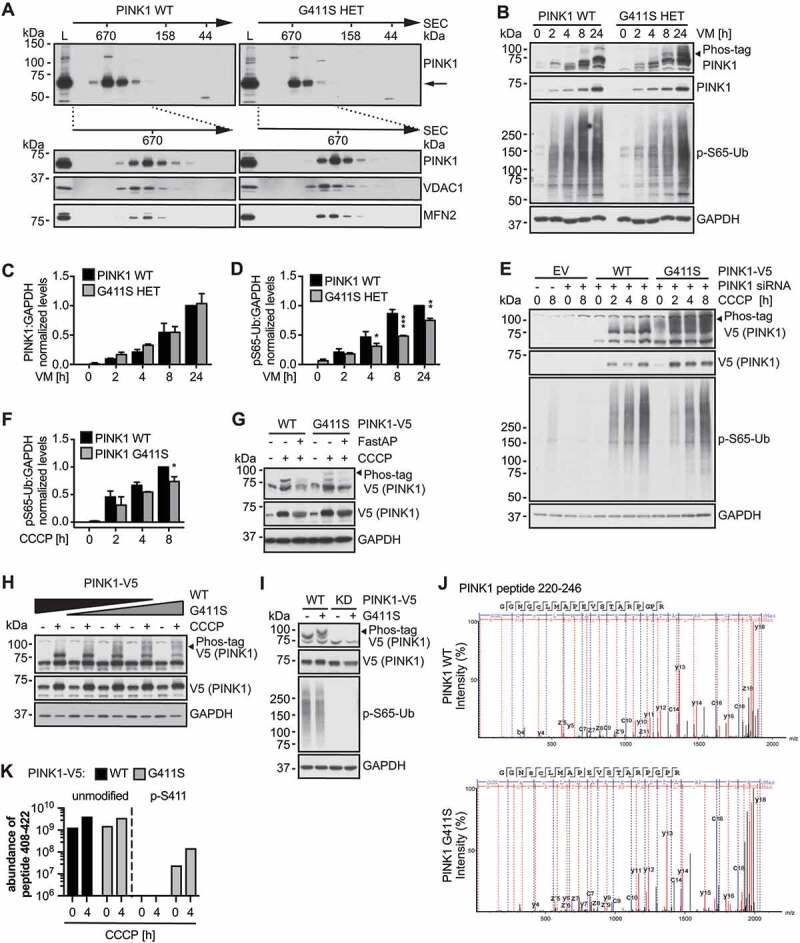
PINK1^G411S^ results in reduced substrate phosphorylation, but increased auto- phosphorylation. (A) Immunoblot analysis of gel filtration (GF) chromatography fractions collected from human wild type (WT) PINK1 and PINK1^G411S^ heterozygous (HET) fibroblast lysates. Molecular weight standards of the GF are indicated on top. Shown is the distribution of full-length PINK1 (indicated by arrow) after treatment with valinomycin (VM) for 16 h. Total cell lysates (L) were run in parallel to pooled (top) or individual GF fractions (bottom). VDAC1 and MFN2 served as controls. (B) Immunoblot analysis of human fibroblasts treated with VM over time. Total lysates were separated by conventional or Phos-tag SDS-PAGE to resolve un-/phosphorylated PINK1 (black arrowhead points to the aberrant autophosphorylation of PINK1^G411S^). (C) Total PINK1 and (D) p-S65-Ub levels were quantified by densitometry and normalized to GAPDH as loading control. (E) HeLa cells were transfected with siRNA to silence endogenous PINK1 and with empty vector (EV) or siRNA-resistant V5-tagged WT or PINK1^G411S^ cDNA. Cells were treated with CCCP for indicated times and lysates were analyzed by conventional and Phos-tag immunoblots. (F) p-S65-Ub levels were quantified by densitometry relative to GAPDH. (G) HeLa cells were transfected with PINK1 siRNA and WT PINK1or PINK1^G411S^ V5-tagged constructs and treated with CCCP for 3 h. Incubation of samples with alkaline phosphatase (FastAP) resulted in collapse of the bands separated by Phos-tag. (H) Different amounts of V5-tagged WT and PINK1^G411S^ cDNA were mixed and transfected as indicated, and cells were treated with CCCP for 3 h. (I) WT or PINK1^G411S^ constructs with or without additional kinase-dead (KD) mutation were transfected and cells were treated with CCCP for 3 h. (J-K) Immunoprecipitated V5-tagged WT and PINK1^G411S^ were analyzed by mass spectrometry. Shown are spectra of the 4-h treated samples (J) as well as the abundance of unmodified and Ser411 phosphorylated peptide 408–420 in log scale (K). (C, D, F) Shown is the normalized mean ± SD from three independent experiments. Statistical significance was assessed with two-way ANOVA and Sidak’s post hoc test (* p < 0.05, ** p < 0.005, *** p < 0.0005).

To follow up on the pathogenic mechanism of the G411S variant, we set out to further characterize and compare PINK1 protein from control and PD patient fibroblasts. PINK1 is cleaved in healthy mitochondria, but upon damage, full-length protein accumulates into a supermolecular complex [3,4,7,9]. Size exclusion chromatography of lysates from challenged fibroblasts confirmed a comparable incorporation of both endogenous WT and PINK1^G411S^ mutant into a higher molecular weight assembly (Figure 1A). However, levels of p-S65-Ub remained significantly lower in PINK1^G411S^ cells compared to WT control fibroblasts (Figure 1B-D). To analyze PINK1 autophosphorylation, which is known to be critical for enzymatic activity [5,6,17], we prepared Phos-tag gels that retard and thereby separate modified from non-modified species. Surprisingly, we noticed additional phosphorylated PINK1 bands in patient cells with the heterozygous PINK1^G411S^ variant compared to WT controls, despite comparable levels of total PINK1 protein (Figure 1B).

Knockdown of endogenous PINK1 in HeLa cells together with low level re-transfection of V5-tagged WT or PINK^G411S^ cDNA further confirmed an increase in PINK1 phosphorylation accompanied by a reduction in Ub substrate phosphorylation (Figure 1E-F). Phosphatase treatment of the samples led to a complete collapse of the shifted bands, as expected for phosphorylated species (Figure 1G). Moreover, changing the ratio of co-transfected PINK1^G411S^ to WT from 0 to 100%, resulted in a gradual increase of PINK1 phosphorylation (Figure 1H). Introduction of the PINK1^G411S^ variant into a kinase-dead background revealed that PINK1 itself catalyzes the aberrant phosphorylation (Figure 1I). However, further efforts to determine the mode of the autophosphorylation (*in cis* or *in trans* in a dimer) remained inconclusive perhaps due to incompatibility of different epitope tags needed for such experiment (data not shown). Lastly, mass spectrometry of V5-tagged PINK1 immuno-purified from HEK293E *PINK1* knockout (KO) cells confirmed that the activation loop mutation Ser411 itself was indeed phosphorylated (Figure 1J-K).

Taken together, while PINK1^G411S^ was stabilized and incorporated into the supermolecular complex similar to WT protein, it showed reduced Ub substrate phosphorylation, but was additionally autophosphorylated at the mutation site.

### PINK1^G411A^ is a more active Ub kinase

**Figure 2.**
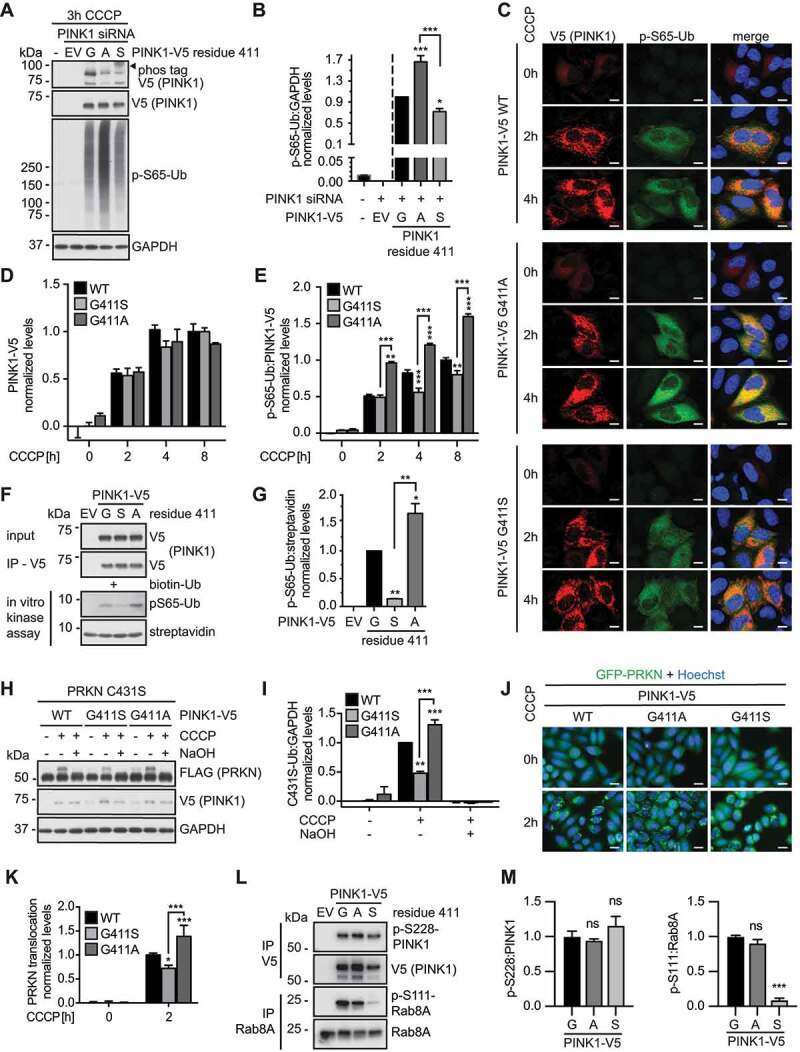
PINK1^G411A^ results in increased Ub substrate phosphorylation. (A) Immunoblot analysis of HeLa cells transfected with PINK1 siRNA and with empty vector (EV), or siRNA-resistant V5-tagged WT PINK1, PINK1^G411A^, or PINK1^G411S^, and treated with CCCP as indicated. (B) p-S65-Ub levels were quantified by densitometry. Shown is the normalized mean ± SD of three independent experiments. (C-E) High content imaging (HCI) analysis of HeLa cells transiently transfected with *PINK1* siRNA and siRNA-resistant V5-tagged WT PINK1, PINK1^G411S^, or PINK1^G411A^ constructs. Cells were left untreated or treated with CCCP for the indicated times, and then fixed and stained with anti-V5 (PINK1, red) and anti-p-S65-Ub (green) antibodies. Nuclei were labeled with Hoechst 33342 (blue). (C) Representative confocal images are shown for all PINK1 variants and time points. Scale bars: 10 µm. HCI quantification of PINK1-V5 (D) and p-S65-Ub (E) signal normalized to the respective PINK1-V5 levels for each individual cell are presented as mean ± SEM. (F, G) *In vitro* kinase assays with PINK1-V5 immunoprecipitated from HeLa cells and biotinylated Ub as substrate. (F) Immunoblot analysis of the reactions using V5 and p-S65-Ub antibodies as well as streptavidin-HRP as a loading control. (G) p-S65-Ub levels normalized to total biotinylated Ub are shown as mean ± SD. (H, I) Immunoblot analysis of Ub-charging of the E3 ligase PRKN. HeLa stably expressing 3xFLAG-PRKN^C431S^ were transiently transfected with PINK1 siRNA and siRNA-resistant V5-tagged WT PINK1, PINK1^G411S^, or PINK1^G411A^ constructs, and were left untreated or treated with CCCP for 1 h. (H) PRKN activation was then assessed by anti-FLAG and an 8-kDa band shift that collapses upon treatment with NaOH. (I) The level of Ub-charging of PRKN is shown as the normalized mean ± SD of three independent experiments. (J, K) HCI and quantification of PRKN translocation to damaged mitochondria. HeLa stably expressing GFP-PRKN were transiently transfected with PINK1 siRNA, mCherry expressing vector and siRNA-resistant V5-tagged V5 WT PINK1, PINK1^G411S^, or PINK1^G411A^. (J) Shown are representative merge images with green GFP-PRKN epifluorescence and nuclear Hoechst signal in blue. Scale bars: 20 µm. (K) Values represent the normalized mean ± SD of four independent experiments. (L, M) V5-tagged PINK1 WT, PINK1^G411A^ or PINK1^G411S^ were transfected into *PINK1* KO HEK293E cells. PINK1 was immunoprecipitated with V5 and RAB8A with RAB8A antibodies. Precipitates were loaded onto a gel and immunoblots were probed with phospho-specific and total antibodies for PINK1 and RAB8A, respectively. Three independent experiments were quantified. Statistical significance was assessed with one-way (B, G, M) or two-way (D, E, I, J) ANOVA and Tukey’s post hoc test (* p < 0.05, ** p < 0.005, *** p < 0.0005). Asterisks on top of data points indicate statistical difference to WT PINK1, while comparison between PINK1^G411A^ and PINK1^G411S^ is indicated by brackets.

Most kinases self-activate by phosphorylation within their activation segment which typically stabilizes this region and imposes conformational changes that promote activity [25]. However, the PINK1^G411S^ mutation introduces a novel autophosphorylation site into the activation loop that diminishes PINK1 kinase activity toward its substrate Ub. In stark contrast, introduction of a non-phosphorylatable PINK1^G411A^ variant resulted in significantly higher levels of Ub phosphorylation compared to PINK1 WT (Figure 2A-B). Puzzled by these findings, we systematically introduced other amino acids at position 411 and tested their activity in CCCP treated cells in comparison to PINK1 WT **(Fig. S1)**. p-S65-Ub levels increased when G411 was substituted with a short nonpolar amino acid such as alanine or valine but decreased then with increasing size of the nonpolar side chain (**Fig. S1A**). Polar substitutions such as PINK1^G411S^ all significantly reduced PINK1 activity with longer side chains having a more drastic effect (**Fig. S1B**). Charged amino acids at position 411 such as aspartic and glutamic acid that sometimes can serve as phospho-mimetic mutants almost completely abrogated p-S65-Ub levels (**Fig. S1C**). Of all substitutions tested PINK1^G411A^ was the most active, whereas the PD mutation PINK1^G411S^ and even more so its phospho-mimetic form negatively impact Ub kinase function.

In order to characterize the effects of PINK1^G411A^ and compared to PINK1^G411S^ in more detail, we next performed high-content imaging (HCI) of PINK1-V5 and p-S65-Ub immunofluorescence staining on the single cell level over time (Figure 2C). While the average V5 expression levels were similar for all variants, PINK1^G411S^ showed significantly lower and PINK1^G411A^ significantly higher levels of p-S65-Ub compared to PINK1 WT (Figure 2D-E). To directly assess enzymatic activities of the PINK1 variants we performed *in vitro* kinase assays with PINK1-V5 immunoprecipitated from cells (Figure 2F-G). Incubation of PINK1-V5 immunoprecipitates with recombinant monomeric Ub confirmed that the substrate kinase activity of PINK1^G411S^ was significantly lower, while that of PINK1^G411A^ was significantly higher compared to PINK1 WT. To determine effects on the pathway further downstream, we performed additional experiments using well-established cell models and paradigms [28]. First, we analyzed Ub-charging of PRKN using its active site mutant PRKN^C431S^ that forms a stable oxyester bond with Ub upon activation of the E3 ligase (Figure 2H-I). Indeed, PINK1^G411S^ showed significantly reduced, while PINK1^G411A^ showed significantly increased Ub-charging of PRKN in comparison to PINK1 WT. As an additional readout for PRKN activation, we assessed its mitochondrial translocation by HCI (Figure 2J-K). Consistently, and compared with WT, PINK1^G411S^ impaired recruitment to damaged mitochondria, whereas the hyperactive PINK1^G411A^ variant increased translocation and activation of the E3 Ub ligase PRKN.

To test potential mechanisms of PINK1^G411A^ and PINK1^G411S^, we investigated the autophosphorylation of PINK1 at S228, which plays a crucial role in enabling the Ub kinase activity of PINK1 [7,9]. We used HEK293E *PINK1* KO cells to express and immunoprecipitate WT and mutant PINK1-V5 after CCCP treatment. A phospho-specific antibody showed that all three proteins were strongly phosphorylated at S228 and the ratio of autophosphorylated to total PINK1 was similar between WT, PINK1^G411A^, and PINK1^G411S^ (Figure 2L-M). Mass spectrometry confirmed these results, as the abundance of the p-S228 phosphorylated peptide was similar among all PINK1-V5 immunoprecipitates **(Fig. S2**). This suggests an effect of residue 411 downstream of PINK1 activation. As an additional readout for PINK1 activity we monitored the phosphorylation of RAB8A at Ser111 [29]. Endogenous RAB8A was successfully immunoprecipitated from PINK1-V5 transfected HEK293E *PINK1* KO cells. While the extent of p-S111-RAB8A to total RAB8A was similar between WT and PINK1^G411A^ at the analyzed time point, PINK1^G411S^ showed strongly reduced phosphorylation of this indirect PINK1 substrate (Figure 2L-M).

In summary, while PINK1^G411S^ and its aberrant autophosphorylation reduced Ub kinase activity and RAB8A phosphorylation, PINK1^G411A^ strongly enhanced Ub phosphorylation with sustained effects on PRKN activation and recruitment to damaged mitochondria. Of note, for both mutations our data suggest mechanisms independent of PINK1 stabilization on mitochondria and autophosphorylation at S228.

### Residue 411 of PINK1 modifies kinase activity under endogenous conditions

**Figure 3.**
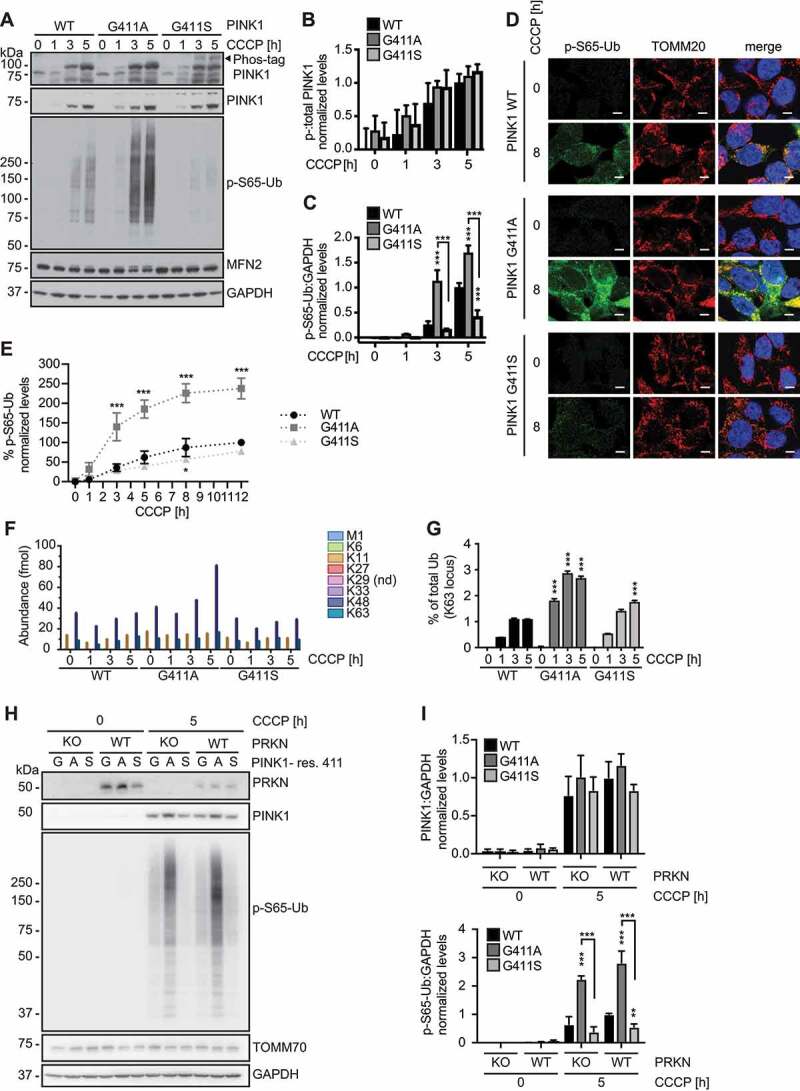
PINK1^G411A^ gene-edited cells show enhanced Ub kinase activity. (A-C) Immunoblot analysis of gene-edited HEK293T cells expressing endogenous WT PINK1, PINK1^G411A^, or PINK1^G411S^. Cells were treated with CCCP for the indicated times and lysates were analyzed by conventional or Phos-tag SDS-PAGE. A black arrowhead points to the aberrant autophosphorylation of PINK1^G411S^. (A) Representative immunoblots are shown alongside densitometric quantification of (B) phosphorylated PINK1 and (C) phosphorylated Ub as normalized mean ± SD from three independent experiments. (D) For immunofluorescence analysis, cells were seeded on glass coverslips, left untreated or treated with CCCP and stained with antibodies against p-S65-Ub (green) and TOMM20 (mitochondria, red). Nuclei were stained with Hoechst 33342 (blue). Scale bars: 5 µm. (E) p-S65-Ub levels were measured by Meso Scale Discovery ELISA. Shown is the mean ± SD from four independent time course experiments. Curves showed significant differences: WT vs. G411A p < 0.0001; WT vs. G411S p = 0.01. (F, G) Targeted, quantitative proteomics of total Ub and p-S65-Ub from mitochondrial fractions of three independent experiments. (F) Absolute quantification (AQUA) of Ub was achieved using heavy reference peptides to determine the individual Ub linkages found on mitochondria at the given times in each cell line. Values shown for each linkage type were normalized to total Ub. Abundant Ub species included K11 (Orange), K48 (dark blue), and K63 (turquoise). (G) p-S65-Ub levels were quantified and are expressed as percent of total Ub (K63). (H, I) Immunoblot analysis and quantification of lysates from gene-edited HEK293T with or without endogenous PRKN expression. Cells were transduced with a lentivirus expressing Cas9 and a guide RNA targeting the start codon of the PRKN gene followed by antibiotic selection. Cells were then left untreated or treated with CCCP side-by-side with the respective parental cells. (B, C, E, G, I) Statistical analysis was performed with two-way ANOVA and Tukey’s post-hoc test. Asterisks on top of data points indicate statistical difference to WT PINK1, while comparison between PINK1^G411A^ and PINK1^G411S^ is indicated by brackets (* p < 0.05, ** p < 0.005, *** p < 0.0005).

To further confirm our findings and the critical role of residue 411 on the endogenous protein level, we introduced the PINK1^G411S^ and PINK1^G411A^ variants into the genome of HEK293T cells using CRISPR/Cas9. Upon CCCP treatment, PINK1 protein stabilized swiftly and to a similar extent across all three genotypes (Figure 3A-B). Phos-tag immunoblots confirmed the additional phosphorylated band specific to PINK1^G411S^, while autophosphorylation of WT and PINK1^G411A^ was comparable. P-S65-Ub was induced earlier in PINK1^G411A^ cells and levels remained significantly higher throughout the time course, while levels were consistently lower in PINK1^G411S^ cells compared to PINK1 WT (Figure 3A, and C). Greater and earlier p-S65-Ub induction in PINK1^G411A^ cells seemed to be accompanied by degradation of the PRKN substrate MFN2.

Immunofluorescence analysis further confirmed the much stronger p-S65-Ub signal on damaged mitochondria in PINK1^G411A^ cells, and the decreased accumulation observed in PINK1^G411S^ cells compared to WT controls (Figure 3D). We also employed a Meso Scale Discovery sandwich ELISA [30] to quantify the relative amounts of p-S65-Ub upon mitochondrial depolarization (Figure 3E). In cells encoding PINK1^G411A^, p-S65-Ub levels were about 2–3 times higher upon CCCP treatment, while PINK1^G411S^ cells showed a modest but consistent and significant reduction over time compared to WT. As an alternative readout, we analyzed mitochondrial fractions in a targeted proteomics approach for an absolute quantification (AQUA) of total Ub and the percentage of p-S65-Ub on mitochondria [31] (Figure 3F-G). While there was no difference in absolute Ub levels on mitochondria before depolarization, we found a substantial increase of especially K48-linked Ub over time in the presence of PINK1^G411A^. We were also able to confirm the increased levels of p-S65-Ub on mitochondria in PINK1^G411A^ cells, both of which were consistent with an enhanced activation of the PINK1-PRKN mitophagy pathway.

To ascertain the “pure” PINK1 kinase effect of residue 411, we sought to uncouple the known feedforward loop between both enzymes through which PRKN can amplify p-S65-Ub levels [20]. We knocked out PRKN in all three cells lines and compared them back to the parental WT, PINK^G411A^, or PINK1^G411S^ cells with endogenous expression of the E3 Ub ligase (Figure 3H). In line with rather low expression levels in HEK293T cells, deletion of PRKN only moderately reduced the appearance of p-S65-Ub upon CCCP treatment. Yet the observed differences between the three PINK1 genotypes persisted, suggesting that the respective p-S65-Ub signals are produced largely by PINK1 alone, independent of PRKN (Figure 3I).

Altogether, we were able to confirm our findings also on the endogenous level in support of a direct effect caused by a single amino acid exchange in the activation loop of PINK1. Our analyses further suggest a mechanism modifying the Ub kinase downstream of the initial PINK1 activation and independent of PRKN.

### Enhanced Ub phosphorylation through PINK1^G411A^ in midbrain-derived neurons

Encouraged by the striking biochemical phenotype of the PINK1^G411A^ variant in HEK293T cells, we sought to confirm the phenotype in a biologically relevant model. We selected ReN cell VM, a human neural progenitor cell (NPC) line that can be terminally differentiated into dopaminergic neurons [32,33] **(Fig. S3A-B)**. Side-by-side comparison of undifferentiated NPCs and differentiated neurons treated with CCCP revealed a quite striking difference in protein levels of PRKN, but similar amounts of PINK1 **(Fig. S3C-D)**. While phosphorylation of Ub was detectable in NPCs upon mitochondrial damage, much greater levels of p-S65-Ub were induced in differentiated neurons, most likely due to the higher expression of PRKN. This was also reflected by a larger decline in levels of mitochondrial PRKN substrates over time in differentiated neurons.

**Figure 4.**
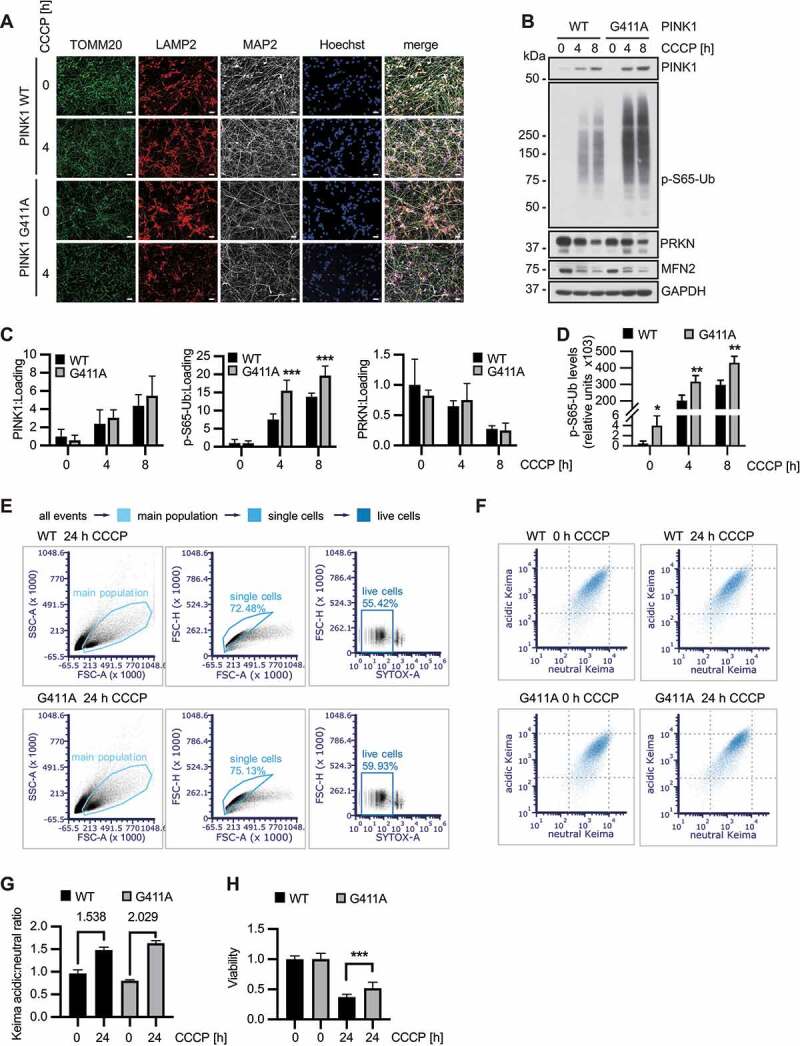
PINK1^G411A^ variant promotes mitophagy in human neurons. (A) Differentiation of NPCs into neurons leads to neuron-like morphology accompanied by the presence of neuronal marker (MAP2). Mitochondrial and lysosomal morphology and distribution seem unchanged in PINK1^G411A^ versus WT cells also at higher magnification (not shown). Scale bars: 10 µm. (B) Immunoblot analysis of isogenic neurons highlights the stronger induction of p-S65-Ub in PINK1^G411A^ cells compared to parental WT cells. (C) Quantification of signals from four independent experiments as shown in (B) confirms statistically significant increase of p-S65-Ub, but not PINK1 or PRKN levels. (D) Lysates from the same experiments were also used for sandwich ELISA, demonstrating significantly higher p-S65-Ub levels even at basal, non-treated conditions. (E-G) Cells were differentiated and the mitoKeima signal measured by flow cytometry. Doublet and dead SYTOX Red positive cells were excluded from the analysis (E) and the acidic and neutral mitoKeima signal was measured from at least 20,000 cells per experiment (F). The geometric mean of the ratio of acidic to neutral Keima ± SD is shown from three independent experiments (G). PINK1^G411A^ neurons show a greater increase of mitophagy compared to WT. (H) Cells were differentiated in 96-well plates and treated with CCCP to induce mitochondrial damage. Viability was determined using CyQUANT. Overnight incubation led to more cell death in wild-type cells compared to PINK1^G411A^, which were partially protected (n = 12). (C, D, H) Shown are the means ± SD of independent experiments. Statistical analysis was performed using two-way ANOVA with Sidak’s post-hoc test (C, H) or Student’s two-sided t-test to compare levels at baseline (D) (* p < 0.05, ** p < 0.005, *** p < 0.0005).

Using CRISPR-Cas9, we introduced a homozygous PINK1^G411A^ or a heterozygous PINK1^G411S^ mutation into the genome of ReN cell VM NPCs **(Fig. S3E)**. Successful gene-editing and absence of off-target modifications was confirmed by Sanger sequencing **(Fig. S3F)**. For proof of concept, cell clones were immediately analyzed for levels of PINK1 and p-S65-Ub. As expected, introduction of the PINK1^G411A^ mutation resulted in a robust increase of p-S65-Ub levels even in undifferentiated NPCs with relatively low PRKN expression **(Fig. S3G)**. Consistent with observations from the various non-neuronal cells, introduction of a single PINK1^G411S^ mutation noticeably reduced p-S65-Ub levels in differentiated neurons **(Fig. S3H)**. Further no viability issues were noted in undifferentiated NPCs or differentiated neurons and the latter were consistently stained with the neuronal marker MAP2. There were also no obvious effects on differentiation or neurite outgrowth under basal conditions. Likewise, mitochondrial and lysosomal staining did not show overt differences between the genotypes (Figure 4A). A detailed analysis of differentiated neurons by immunoblot confirmed a significant increase of p-S65-Ub for PINK1^G411A^, with no change in PINK1 or PRKN levels in these cells compared to WT (Figure 4B-C). Data obtained with a sensitive p-S65-Ub Meso Scale Discovery ELISA [30] further suggested that p-S65-Ub levels were slightly, but significantly elevated in PINK1^G411A^ even at baseline in untreated neurons (Figure 4D). To determine mitophagy rates in these neurons, we employed flow cytometry as a highly reproducible and sensitive method to quantify a large number of cells. Careful gating of single and live cells was followed by analysis of the lysosomal (acidic) to mitochondrial (neutral) Keima signal for each individual cell of at least 20,000 cells per experiment (Figure 4E-F). After 24 h CCCP treatment, there was a greater induction of mitophagy in PINK1^G411A^ neurons compared to WT (Figure 4G). In addition, of the neurons incubated with the depolarizer, those expressing PINK1^G411A^ showed overall an increased viability (Figure 4H) pointing to a potential neuroprotective effect exerted by this more active PINK1 variant.

Together our findings confirm the strongly enhanced Ub kinase activity of PINK1^G411A^ under endogenous conditions and in the context of disease-relevant dopamine neurons. Moreover, our data emphasize the critical role of PRKN in further amplification of the p-S65-Ub signal and the resulting increased turnover of damaged mitochondria that seems to provide some level of resilience to neurons.

### Model of the human PINK1 suggests residue 411 influences substrate receptivity and S65 positioning of Ub

**Figure 5.**
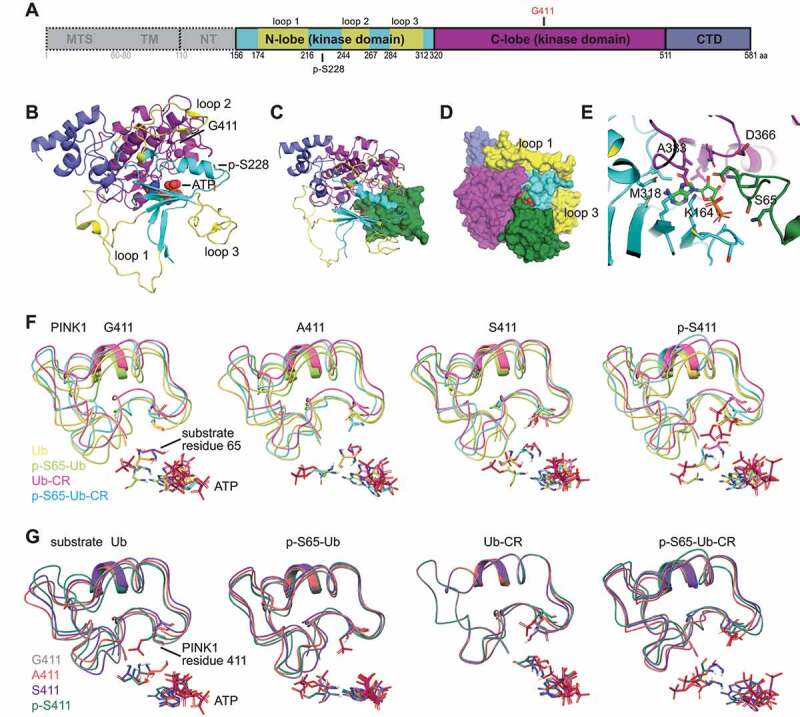
PINK1^G411A^ improves relative positioning of ATP and substrate residue S65 within the active site. (A) Schematic representation of the entire human PINK1 protein including the mitochondrial targeting sequence (MTS), transmembrane region (TM) and N-terminal region (NT). Individual domains of the catalytically active fragment (aa 156–581) studied are color-coded: N- and C-lobes of the kinase domain in cyan and magenta, respectively; C-terminal domain (CTD) in blue. The three unique insertion loops are highlighted in yellow. Position 411 and the autophosphorylation site S228 are indicated. (B) 3D representation of the human PINK1 model with ATP shown in spheres. (C) 3D representation of the human PINK1 model with Ub bound shown as a green Connolly surface and ATP in stick representation. (D) The model is rotated 90° about the 45° -y/+x bisector, relative to (C). All components are represented as Connolly surfaces to indicate optimal packing was achieved. (E) Close-up of the PINK1, ATP, and Ub interface, with interacting residues represented as sticks. (F-G) Overlay of reduced representations of the different enzyme-substrate combinations with matching structures color-coded and aligned by (F) PINK1 variant or (G) substrate S65. PINK1 residues C388-Y431 containing the activation segment are depicted as ribbons and position 411, ATP, and S65 of the Ub substrates are shown in licorice stick rendering. (F) Positioning of S65 relative to WT PINK1, PINK1^G411A^, PINK1^G411S^, and PINK1^p-^^S411^ are shown from left to right and color-coded according to the substrates: Ub (yellow), p-S65-Ub (lime green), Ub-CR (magenta), and p-S65-Ub-CR (cyan). For PINK1^G411A^, note the alignment for ATP and the oxygen atom of S65 of both Ub and Ub-CR. (G) Positioning of PINK1 and residues 411 relative to the respective S65 residues of Ub, p-S65-Ub, Ub-CR, and p-S65-Ub-CR are shown from left to right and color-coded according to the PINK1 variant: G411 (gray), A411 (salmon), S411 (purple), and p-S411 (olive). For Ub-CR, note the almost identical overlap for the activation segments of all PINK1 variants.

To shed more light onto the role of residue 411 and potential mechanisms for modified Ub phosphorylation, we turned to computational modeling. Incorporating recent structural information from insect PINK1 orthologs [21–23] we improved our earlier model of human PINK1 protein [27] (Figure 5A-B). We phosphorylated the critical Ser228 residue *in silico* and focused on the soluble fragment of PINK1 containing the activated Ub kinase (residues 156–581). A detailed domain-by-domain analysis of the various structures can be found in **Fig. S4**. To assess substrate binding and positioning, we docked Ub to the autophosphorylated kinase domain of human PINK1 (Figure 5C-E) resulting in similar orientation and distances regarding N-lobe, insertion loop 3, and activation segment and also matching some of the reported interactions of Ub and insect PINK1 [23]. We then created and compared models for each PINK1 variant: WT (G411), PINK1^G411A^, PINK1^G411S^, and PINK1^p-^^S411^, with the additional autophosphorylation in the activation segment. It has been shown that the preferred substrate of PINK1 is not the common conformation of Ub (PDB: 1UBQ), but a minor (<1%), C-terminally retracted form (CR) with an extended S65 containing loop (PDB: 6EQI) [23,26]. Given this selectivity, we docked different Ub substrates including the common Ub and the minor Ub-CR conformation as well as their phosphorylated counterparts (p-S65) to each of the PINK1 variant models. An overview of the 16 different substrate-enzyme complexes can be found in **Fig. S5**.

For direct comparison, we first overlaid regions containing the activation segments of the four PINK1 variants (residues 388–431) and determined the orientation of the substrate S65 residues with regard to ATP (Figure 5F). As expected, PINK1 WT showed an improved positioning of the target S65 of Ub-CR compared to regular Ub, while the two phosphorylated versions were less well-aligned. Of note, PINK1^G411A^ revealed an identical position of the S65 oxygen atom for both Ub and Ub-CR. In contrast, for PINK1^G411S^, S65 of Ub was less well-aligned with ATP and the entire activation loop seemed to retract even further in the PINK1^p-^^S411^ model likely to the negative charge of the phosphorylation therein. Changing the perspective, we overlaid all four Ub substrates and looked at the relative position of the PINK1 activation segments containing either G411, G411A, G411S, or p-S411 (Figure 5G). We observed that upon docking of Ub to PINK1^G411A^ there was more overall surface-to-surface contact compared to WT, while both PINK1^G411S^ and its phosphorylated form had less interaction with Ub. Yet, for Ub-CR, the activation loops of all four PINK1 variants were almost entirely matching. It appeared that this minor Ub conformation induced rigidity of the PINK1 activation loop, which in turn might help improve binding. In contrast, both phosphorylated Ub substrates were shifted sideways and less well aligned between the activation loop and ATP. These observations were further supported by calculations of free energy perturbation (FEP+), proximity, and interface areas between the PINK1 variants and the respective Ub substrates **(Table S1)**.

Taken together, PINK1^G411A^ showed a more favorable alignment for S65 from the common Ub structure suggesting that the mutant kinase may accept both substrate conformations similarly well.

### G411A stabilizes the PINK1 structure and induces the minor conformation of Ub

**Figure 6.**
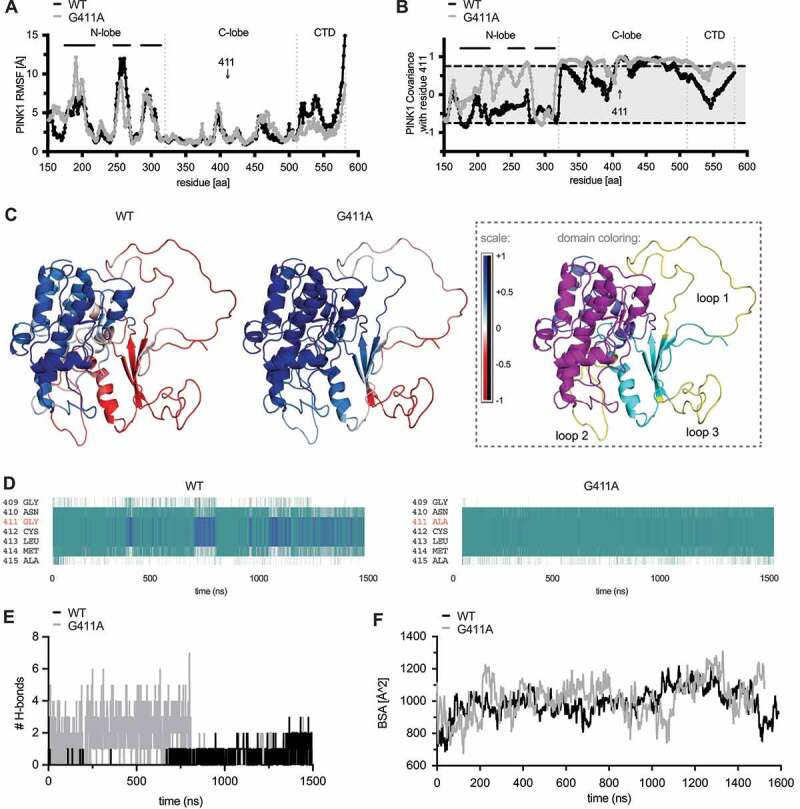
PINK1^G411A^ induces conformational changes that stabilize the kinase fold. (A) Root mean square fluctuations (RMSF) of backbone atoms over time are shown for residues 156–581 of WT PINK1 (black) and PINK1^G411A^ (gray). Vertical dotted lines separate the individual (sub)domains of PINK1 and horizontal solid lines highlight the 3 unique insertion loops in the N-lobe domain. The position of residue 411 is indicated with an arrow. (B) Analysis of correlated motions between position 411 and all other residues in the catalytically active fragment of WT PINK1 (black) and PINK1^G411A^ (gray). Grcarma was used to generate the depicted mass-weighted, normalized covariance matrices. PINK1 (sub)domains and residue 411 are indicated as above in (A). All highly significant correlated motions are found above or below the gray shaded area (≧ +0.75 or ≦ 0.75). (C) Heatmap presentation of correlated motions mapped onto the ribbon structures of WT PINK1 (left) and PINK1^G411A^ (right) highlights the strong coordination of motions within and between the kinase subdomains of the variant. The structures are colored on a five-point covariance scale from dark (−1.0) and light red (−0.5) to white (0) to light (+0.5) and dark blue (+1.0). (D) Analysis of the PINK1 secondary structure was carried out for every frame across the duration of the simulation using the Timeline module of VMD. Provided are excerpts from the entire analysis focusing only on the immediate region surrounding residue 411 for WT PINK1 (left) and PINK1^G411A^ (right) which showed a reduction of 3–10 helices (blue) but an increase of turns (teal) over time. (E) Calculation of the number of H-bonds formed between insertion loop 3 and Ub over time for each simulation shows greater interactions between Ub and PINK1 compared to WT PINK1. (F) Assessment of the buried surface area (BSA) of the PINK1-Ub complex over time for each simulation shows a slightly higher average and greater maximum BSA for PINK1^G411A^ compared to WT PINK1.

To characterize potential structural changes over time, we performed relatively long (1.5 μsec) molecular dynamics simulations (MDS) of WT and PINK1^G411A^, each in complex with ATP and normal Ub (i.e., the common conformation). Root mean square deviations (RMSD) of atomic positions across the duration of the simulation were only subtle for all structures **(Fig. S6A-B)**. We first focused on intrinsic motions in PINK1 (both globally and locally), starting with an analysis of the root mean square fluctuations (RMSF) per residue over time (Figure 6A). Differences between WT and PINK1^G411A^ were notable in the N-lobe, where fluctuations seemed especially prominent within the unique insertion loops 1 and 2, with no major changes seen in loop 3. Additional differences between both structures were found in the CTD region. Except for residues from insertion loop 1, the spatial extent of random, uncoordinated motion was generally reduced in PINK1^G411A^ compared to WT. We next investigated correlated motions based on mass-weighted, normalized covariance matrices. Interestingly, the PINK1^G411A^ variant seemed to promote coordination between N- and C-lobes of the kinase domain as well as within the C-lobe itself (Figure 6B-C). Finally, we inspected the secondary structure with a particular focus on the region surrounding residue 411, which exists as either a turn or a 3–10 helix for WT PINK1 (80.98% vs. 19.02% of the time, respectively). (Figure 6D). For PINK1^G411A^, the turn was further increased while the 3–10 helix was reduced (98.18% vs. 1.82% of the time, respectively), which may allow for more regular propagation of motion within and between the two lobes of the kinase domain. However, the number of H-bonds formed between loop 3 and Ub over time was greater for PINK1^G411A^ than WT (Figure 6E) as was the buried surface area of the PINK1:Ub complex (Figure 6F) both of which suggest more favorable interaction between kinase and substrate.

**Figure 7.**
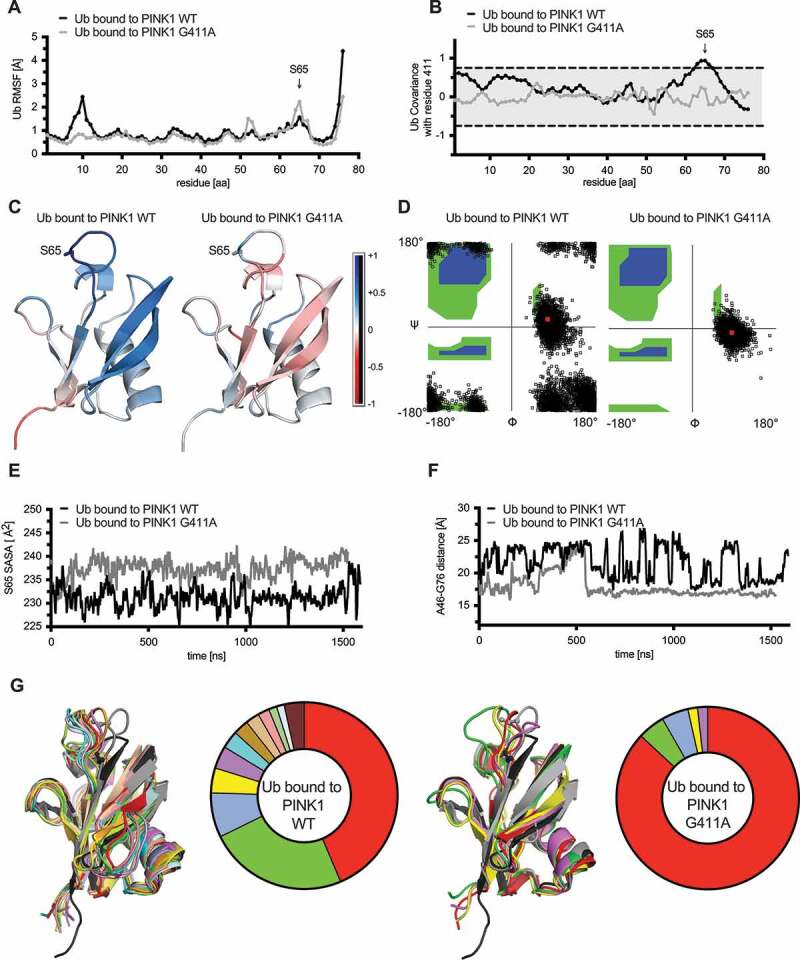
Ub bound to PINK1^G411A^ adopts a C-terminal retracted conformation. (A) RMSF are shown for the substrate Ub bound to either WT PINK1 (black) or PINK1^G411A^ (gray). Residue S65 of Ub is highlighted with an arrow. (B) Analysis of correlated motions between position 411 of PINK1 and all residues within the substrate Ub bound to WT PINK1 (black) or PINK1^G411A^ (gray). (C) Heatmap presentation of correlated motions mapped onto the Ub structures bound to WT PINK1 (left) or PINK1^G411A^ (right) highlights the reduced covariance of Ub in complex with the PINK1 variant. The structures are colored on a five-point covariance scale from dark (−1.0) and light red (−0.5) to white (0) to light (+0.5) and dark blue (+1.0). (D) Shown are distributions of all Ramachandran angles adopted by Gly10 during the simulation of Ub bound to WT PINK1 (left) or PINK1^G411A^ (right). The Ramachandran angles of Gly10 from either the common (1UBQ) or the minor Ub conformation (6EQI) are represented by the red dots almost centered within the clouds observed for WT PINK1 or PINK1^G411A^, respectively. (E) The solvent accessible surface area (SASA) for S65 was calculated across every frame for the entire simulation of Ub bound to WT PINK1 (black) or PINK1^G411A^ (gray). Shown are the 5 nsec moving average. (F) Proximity measures between Ala46 and Gly76 of Ub bound to WT PINK1 (black) or PINK1^G411A^ (gray). Depicted are the 5 nsec moving average of the distance. (G) Ub conformations were sorted using the Quality Threshold algorithm with heuristically determined RMSD cutoffs to obtain a reasonable number of total clusters, with minimum cluster size approximately 1% of the frames, and rejection rate less than 5%. Shown is an overlay of the center frames of the main clusters for WT PINK1 (left) and PINK1^G411A^ (right) together with the common Ub conformation (1UBQ) in black and the minor Ub-CR structure (6EQI) in gray. Ser65 is shown in ball and stick model for these structures as a reference point. The center frame of each unique cluster was subjected to energetics calculation using FoldX and the raw score was weighted by the percentage of frames represented by that particular cluster. The color-matched pie chart depicts the frequencies of each cluster. The respective raw and weighted ΔG values of the interaction between PINK1 and Ub from each cluster can be found in Table S2.

We next turned our attention to the conformational changes in Ub that was bound to each PINK1 model during the simulations. Analysis of the RMSF per residue of Ub revealed distinct peaks (Figure 7A) that partially overlapped with important motifs and interaction surfaces of Ub [34]. Compared to PINK1^G411A^, Ub bound to WT PINK1 showed much greater fluctuations in a region spanning residues 6–13 (peak at Gly10) as well as of the C-terminal tail (aa 73–76). In contrast, two other, narrower regions peaking at Asp52 and strikingly at Ser65, were more pronounced in Ub bound to PINK1^G411A^ compared to WT. Correlated motions of all Ub residues with position 411 of PINK1 during the simulation (Figure 7B-C) showed only for WT PINK1 a significant positive correlation with Ub residues immediately surrounding Ser65. A closer look at the secondary structure of Ub and especially at residues 73–76 revealed a turnover 35.14% of simulation time in the context of WT PINK1 **(Fig. S6C)**. However, this increased to 79.41% of simulation time when bound to PINK1^G411A^, further supporting the overall restricted motions and a greatly reduced flexibility of the C terminus of Ub.

Through additional analyses we sought to determine whether these changes were reminiscent of the minor Ub conformation that seems to be the preferred PINK1 substrate [23,26]. First, the Ramachandran angles adopted by residue Gly10, confirmed a greater stability in that region for Ub bound to PINK1^G411A^ with a distribution more similar to the Ub-CR structure (Figure 7D). Second, solvent accessible surface area (SASA) showed a persistently increased exposure of Ser65 when Ub was bound to PINK1^G411A^ (Figure 7E). Third, as a measure of C-terminal retraction, the distance between Ala46 and Gly76 showed relatively large oscillations for Ub when bound to WT PINK1, but this was reduced in the context of PINK1^G411A^ and remained stable for most of the time (last 2/3 of the simulation) (Figure 7F). Last, we categorized all frames (250 psec/frame) of the simulations into distinct conformational clusters and then analyzed and directly compared them back to the reference Ub structures (Figure 7G). Ub not only adopted fewer conformations when bound to PINK1^G411A^, but the majority of these trajectories fell into structural clusters closely resembling Ub-CR. Weighted energetics calculations confirmed an overall more favorable interaction with the respective Ub conformations in case of PINK1^G411A^ (−0.55 kcal/mol) compared to WT PINK1 (1.95 kcal/mol). Raw and weighted ΔG values of interaction between PINK1 and Ub, complex stability, and total potential energy for each cluster can be found in **Table S2**.

Altogether our simulations and comprehensive analyses support a less dynamic PINK1^G411A^ complex that propagates motions to Ub, which leads to a higher frequency in C-terminal tail retraction and a solvent exposed Ser65, which may allow more efficient phosphorylation of the substrate.

### PINK1^G411A^ is more receptive to and efficiently phosphorylates also the common Ub conformation

**Figure 8.**
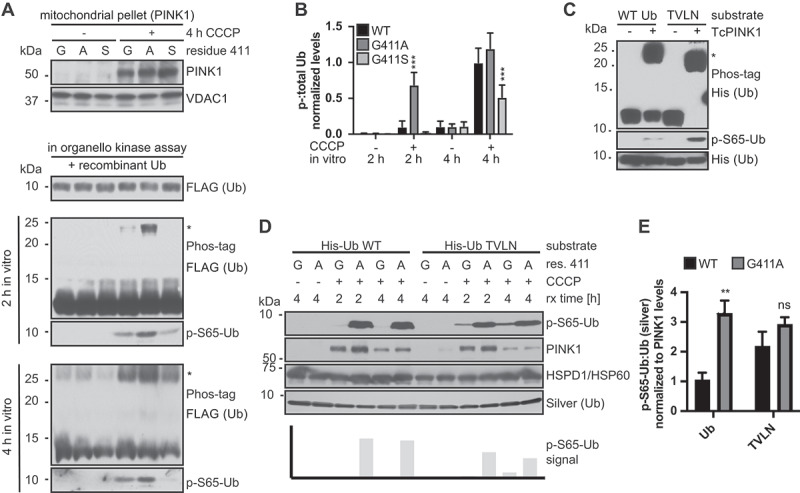
PINK1^G411A^ does not discriminate between the common (WT) and the minor (TVLN) confirmation of Ub. (A) Mitochondrial fractions from HEK293T cells – PINK1^G411^ (G), PINK1^A411^ (A), or PINK1^S411^ (S) – were used for kinase reactions with recombinant FLAG-tagged Ub as the substrate. Supernatants were analyzed and Ub phosphorylation was determined with anti-p-S65-Ub or anti-FLAG antibody using Phos-tag gels (asterisk marks phosphorylated Ub). VDAC1 was used as a mitochondrial loading control. (B) Quantifications of the p-S65-Ub:Ub ratio from Phos-tag gels. Shown is the mean ± SD of three independent experiments. Statistical analysis was performed using two-way ANOVA and Tukey’s post-host test (*** p < 0.0005). (C) In vitro kinase reactions confirm that the Ub[TVLN] (Ub-CR) is a superior substrate for TcPINK1 compared to WT Ub. Recombinant TcPINK1 was incubated with equal amounts of His-tagged WT or Ub[TVLN] for 90 min. Note the substantial amounts of WT Ub that remain un-phosphorylated under these conditions. (D) Mitochondria from HEK293T WT PINK1 (labeled G) and PINK1^G411A^ (labeled A) cells were used for kinase reactions as in (A), with His-tagged WT Ub or Ub[TVLN] as substrate as in (C). HSPD1/HSP60 was used as a mitochondrial loading control, and silver staining to quantify total Ub. Note the strong phosphorylation of WT Ub by PINK1^G411A^, while C-terminally retracted Ub[TVLN] was phosphorylated by both WT PINK1 and PINK1^G411A^. (E) Quantification of *in organello* kinase reactions confirm an overall stronger and equally efficient phosphorylation of WT Ub and Ub[TVLN] by PINK1^G411A^, compared to WT PINK1. Shown is the mean of p-S65-Ub:Ub normalized to PINK1 levels at 4 h reaction time ± SD of five experiments. Statistical analysis was performed using two-way ANOVA with Sidak’s post-hoc test (** p < 0.005).

Enthused by the structural prediction, we turned to our gene-edited cell models again to validate the proposed mechanism by probing substrate selectivity of WT and the PINK1^G411A^ mutant *in vitro*. The field struggles to purify recombinant, active human PINK1, so we isolated mitochondria from CCCP-treated cells of different PINK1 genotypes as a source of the human kinase. As further controls, we used mitochondrial preparations from cells that had been left untreated (i.e. without PINK1). As a defined substrate for these *in organello* kinase assays, we used recombinant monomeric (FLAG-tagged) Ub for different reaction times. With this set up, we were able to confirm the significantly enhanced Ub kinase activity of PINK1^G411A^ compared to WT, and the reduced activity of the G411S mutant (Figure 8A-B), similar to our findings in cells in culture.

Next, we sought to corroborate that the C-terminally retracted Ub conformation, which seems to bind PINK1 with higher affinity, is indeed the preferred substrate of the kinase as previously reported [23,26]. For this we used recombinant *Tribolium castaneum* TcPINK1 protein (which in contrast to human PINK1 can be purified) and directly compared phosphorylation of WT Ub to a Ub variant that adopts the minor, C-terminally retracted conformation regardless of phosphorylation status. This is achieved by introduction of two point mutations (T66V and L67N) and is hereafter referred to as Ub[TVLN] [26] (Figure 8C). Having established a striking difference in phosphorylation of the two substrates, we wanted to determine whether human WT PINK1 or the PINK1^G411A^ variant similarly discriminate between the common and minor Ub conformation. We thus performed side-by-side *in vitro* assays with isolated mitochondria containing human PINK1 and either WT Ub or Ub[TVLN] as the substrates (Figure 8D). Consistent with the structural modeling, PINK1^G411A^ seemed equally receptive to either Ub substrate and effectively and comparably phosphorylated both (Figure 8E). WT PINK1 was overall less active than PINK1^G411A^ but especially less efficient in phosphorylating Ub WT.

In summary, we provide experimental evidence that compared to WT, PINK1^G411A^ is more receptive to and allows efficient phosphorylation of also Ub WT similarly to the C-terminally retracted mutant. This is consistent with the idea from structural prediction that the PINK1 variant may transform the common Ub structure into the more suitable Ub-CR conformation, thereby increasing the abundance of its preferred substrate conformation which ultimately leads to increased turnover.

## Discussion

The PINK1-PRKN directed mitophagy pathway is emerging as a promising future therapeutic avenue with potentially far-reaching implications. The vital roles of both enzymes are well supported by human genetic findings and the broad exacerbating phenotypes in loss of function models. However, direct experimental evidence for their protective effects is scarce and mostly relies on overexpression of PINK1 or PRKN. Likewise, though widely assumed, clear-cut evidence for the beneficial effect of increasing mitophagy is lacking thus far although there are increasing efforts to activate (or derepress) the pathway for potential therapeutic targeting of PD (reviewed in [35]). By probing further into the mechanisms of the pathogenic PINK1^G411S^ mutation, we herein identify a novel, potentially protective variant with strikingly enhanced enzymatic activity compared to WT PINK1. Based on structure-function analyses we propose that PINK1^G411A^ effectively changes Ub receptivity and positioning, and thus expands the actual abundance of the minor conformation of Ub that is the preferred substrate. The resulting increase in Ub phosphorylation is robust and consistently seen across different models and under endogenous conditions in gene-edited cells. Increased levels of p-S65-Ub translate further into augmented PRKN activation and greater mitophagy rates that seem to protect differentiated neurons in culture from mitochondrial stress. Our genetic proof of concept model will lead to a better understanding of the consequences of boosting PINK1-PRKN mitophagy under endogenous conditions. The PINK1^G411A^ variant may not only be useful as a critical positive control but may also help guide the development of small molecule PINK1 activators.

While complete loss of PINK1 or PRKN invariably results in early-onset PD, the contributions of reduced function to disease later in life remains uncertain. The most recent large-scale genetic studies failed to provide unequivocal support for a role of heterozygous mutations as a robust risk factor for PD [36–38]. However, structural and functional analyses have clearly demonstrated that not all mutations are alike. In fact, much has been learned about the regulation and sequence of events along the pathway from missense variants with distinct biochemical or cellular phenotypes [39–45]. Our previous work revealed a sizable contribution of the PINK1^G411S^ mutation to risk and onset of PD caused by a distinctive partial dominant-negative mechanism of the mutation [27,46]. Probing further into the mechanisms of PINK1^G411S^ using primary patient and gene-edited cells, we here confirm and expand our earlier findings. We demonstrate that PINK1^G411S^ is indistinguishable from WT with regard to its stabilization and dimerization into a supermolecular complex upon mitochondrial stress. Yet, we also uncover that PINK1^G411S^ hyper-autophosphorylates at the newly introduced serine residue in the activation loop, which seems to repel the Ub substrate. While most kinases are activated by autophosphorylation in that region [25], PINK1 autophosphorylates at S228 in the catalytic loop which is critical for activation and enables recognition and binding of Ub [3–7,9].

More importantly though, we also identify a more active form of PINK1 here during our investigation. When introducing G411A as a variant, the resulting p-S65-Ub levels were significantly elevated in comparison to WT PINK1, and this was consistently seen across cell types and under endogenous conditions. Despite a potential involvement of G411 in dimerization of PINK1 [7,9], it is noteworthy that we found no evidence that mutations of this residue would alter stabilization or autophosphorylation of PINK1 at S228. Rather our data collectively points to an effect downstream of the initial PINK1 activation consistent with the increased substrate receptivity caused by the transformation of the Ub structure when bound to PINK1^G411A^. Overall we propose that PINK1^G411A^ stabilizes the kinase fold causing Ub to more often adopt the minor, C-terminally retracted conformation, which better exposes Ser65, thereby effectively increasing the abundance of this preferred PINK1 substrate. However, it needs to be clarified, whether the PINK1^G411A^ variant is indeed causing a more effective conversion of the structure of Ub or bypassing the need for this conformational change.

Notwithstanding, what has already become clear is that small upstream effects are amplified along the pathway through the concerted actions of the Ub kinase-ligase pair. While the feedforward loop in which PRKN provides more substrates for PINK1 resulting in further PRKN activation and recruitment is well known, our data further emphasize this could indeed be exploited for therapeutic intervention. When knocking out PRKN in HEK293T cells gene-edited for PINK1^G411A^, p-S65-Ub level were only moderately reduced, consistent with the relatively low endogenous expression of the E3 Ub ligase. Conversely, upregulation of PRKN during differentiation of gene-edited ReN cells from precursor to neurons boosted the p-S65-Ub signal. While precursor and neurons both showed comparable PINK1 protein levels and stabilization over time upon depolarizer treatment, PRKN expression was about 20-fold higher resulting in an about 3–4 fold increase of p-S65-Ub. However, whether PINK1^G411A^ also directly enhances the phosphorylation of Ser65 in the Ub-like domain of PRKN, similar to Ub, cannot be clarified in cell models alone as elevated levels of p-S65-Ub also indirectly lead to increased PRKN activation and phosphorylation by PINK1. Regardless, small molecules mimicking the described G411A effect may be useful to also compensate for reduced expression or enzymatic activity of either PINK1 or PRKN to boost mitophagy, for instance in heterozygous mutation carrier at risk for developing disease.

Nevertheless, our study also has clear limitations. Most importantly, and although in excellent agreement with the published insect structures, several of our findings are based on computational predictions of human PINK1. Yet, we performed relatively long molecular dynamics simulations (~1.5 µsec) and do provide experimental data supporting the main conclusion – PINK1^G411A^ can phosphorylate Ub WT (i.e., the common conformation) similarly well to the Ub[TVLN] mutant (reflecting the minor, C-terminally retracted conformation) that is the more suitable substrate for WT PINK1. However, given that human PINK1 has been notoriously difficult to purify, our kinase assays were performed *in organello* – using purified mitochondria from gene-edited cells as the source for human PINK1 – and not reconstituted *in vitro* with pure, recombinant proteins. Future advancements in expression and purification methods should provide larger quantities of human PINK1 needed for critical evaluation and definitive proof of the proposed mechanism. The following experimental structural analyses should be able to inform about Ub positioning and conformation. In this context it might be informative to include additional PINK1 substrates, such as PRKN as well as poly-Ub chains of different lengths and linkages that are “free” or conjugated to a mitochondrial substrate protein of PRKN. Additional biophysical experiments should help establish binding modes and kinetic profiles in combination with the common or the minor Ub conformation (WT Ub or Ub[TVLN] mutant, respectively).

While we have already shown that substitution of G411 with polar or charged amino acids all lead to reduced p-S65-Ub levels, additional mutation scanning in the PINK1 activation segment around residue 411 of human PINK1 could reveal further insights into this novel mechanism of action. Although G411 is conserved in mammals, it is quite divergent in lower organisms such as insects, worms and zebrafish. Notably, *C. elegans* and *D. melanogaster* both carry an alanine, while zebrafish has a serine at position 411. Given these sequence differences it could be interesting to compare PINK1 from these species to human in order to establish a structure-activity relationship also with respect to dimerization and Ub binding. These studies might enable future drug design, in addition to but different from the currently studied ATP analogs [47,48], capitalizing on the transformational changes in Ub receptivity and positioning seen for PINK1^G411A^.

Lastly, while we do provide a genetic model for PINK1 activation, the full consequences and thus potential therapeutic value of enhancing Ub phosphorylation under these endogenous conditions remain to be established. In general support of a beneficial role, we do find a promising increase in mitophagy rates and a concomitant protection of gene-edited neurons upon mitochondrial stress. Yet, to highlight long-term beneficial effects and/or potential issues arising from a more active form of PINK1, it will be essential to carefully characterize mitochondrial health and cellular fitness at both baseline and over time under chronic or acute stress conditions. Beyond the current cell models, studies should extend to more complex and disease-relevant iPSC-derived culture systems. Unbiased investigations of transcriptome, proteome, and metabolome should help flag any obscure alterations. Animal work is eventually needed to exclude potential concerns in the organismal context, within the brain and in peripheral tissues in which PINK1 and PRKN have been shown to play a role [49]. Studies during development and normal aging as well as under different disease conditions should further help de-risk pharmacological efforts and provide additional support for boosting PINK1-PRKN mitophagy as viable therapeutic avenue.

## Materials and methods

### Cell culture

Primary human dermal fibroblasts [27], collected under approved Mayo Clinic ethical review board protocols, and control fibroblasts (Cell Applications Inc., 106–05A) were grown in Dulbecco’s modified Eagle medium (DMEM; Thermo Fisher Scientific, 11965118) supplemented with 10% fetal bovine serum (FBS; Neuromics, FBS001800112), 1% PenStrep (Thermo Fisher Scientific, 15140122) and 1% non-essential amino acids (Thermo Fisher Scientific, 11140050). Parental human cervical carcinoma cells (HeLa; ATCC, CCL-2), stable HeLa cell clones expressing EGFP-PRKN or 3xFLAG-PRKN^C431S^ [28], parental and genetically modified human embryonic kidney cells HEK293E (ATCC, CRL-10852, originally marketed by Invitrogen) and HEK293T (ATCC, CRL-3216) were cultured in DMEM supplemented with 10% FBS. Neuronal progenitor cells (NPCs) derived from the ventral mesencephalon (ReN cell VM; Millipore, SCC008) were maintained on growth factor reduced matrigel (Corning, 354230) coated plates in DMEM-F12 media (Thermo Fisher Scientific, 10565042), supplemented with B27 (Thermo Fisher Scientific, 17504044), 5 U/ml Heparin (Sigma Aldrich, H3149), and 50 µg/ml gentamicin (Thermo Fisher Scientific, 15750060) in the presence of 20 ng/ml fibroblasts growth factor (FGF; Peprotech, 100–18B) and epidermal growth factor (EGF; Peprotech, AF-100-15). Differentiation of ReN cells was performed by substituting FGF and EGF with 1 mM dibutyryl-cAMP (Invivochem, V1846) and 2 ng/ml GDNF (Peprotech, 450–10) for at least seven days [33]. All cells were grown at 37°C, 5% CO_2_:air in a humidified atmosphere.

### Chemical treatments and transient siRNA and DNA transfection

Carbonyl cyanide m-chlorophenyl hydrazone (CCCP; Sigma Aldrich, C2759) was used at a concentration of 10 µM for all experiments with HEK293 and HeLa and 20 µM for all ReN cell experiments. Valinomycin (Cayman Chemical, 10009152) was used for experiments with fibroblasts at 1 µM. HeLa cells were plated 20–24 h before transfection with siRNA/DNA using Lipofectamine 2000 (Thermo Fisher Scientific, 11668019). The following amounts were used for transient siRNA and DNA transfection per 6-well: 3 µl of 20 nM siRNA, 500 ng of *PINK1* cDNA, and 4.5 µl of Lipofectamine per 250 µl of Opti-MEM media (Thermo Fisher Scientific, 51985034). For HCI, media was replaced after 4 h and cells were cultured for an additional 20 h. For immunoblot analysis, media was replaced after one day. Control siRNA (all stars negative control, Qiagen, 1027281) or *PINK1*-specific siRNA (5’-GACGCTGTTCCTCGTTATGAA-3’, Qiagen) were used along with siRNA-resistant *PINK1* cDNA. PINK1 constructs with C-terminal V5 or mCherry tag have been described previously [27]. PINK1 G411 substitutions were introduced by site-directed mutagenesis of WT or KD (kinase dead: K219A+D362A+D384A) *PINK1* cDNA constructs and confirmed by sequencing.

### Size exclusion chromatography

Human fibroblasts were treated with valinomycin for 16 h, lysed by sonication in lysis buffer (50 mM HEPES-NaOH, pH 7.5, 150 mM NaCl, 10 mM KCl, 1 mM EDTA, 0.5 mM EGTA, 1.5 mM MgCl_2_, 10% glycerol, 0.2% NP-40 [Sigma-Aldrich, I3021]) supplemented with protease and phosphatase inhibitors (Roche Applied Science, 11697498001, 04906837001). Samples were diluted to 500 µg total soluble proteins/200 µl with a gel filtration soluble phase (70 mM potassium phosphate, pH 7.2, 100 mM NaCl, 1 mM EDTA) and separated using an Akta Explorer system (GE Healthcare, Boston, MA, USA). A TSKgel G3000SWXL gel-filtration column (Sigma-Aldrich, 808541) was used at room temperature and calibrated using molecular mass standards TG (thyroglobulin) 670 kDa, γ-globulin 158 kDa, SERPIN (ovalbumin) 44 kDa, MB (myoglobin) 17 kDa, vitamin B_12_ 1.4 kDa (Bio-Rad, 1511901). The elution rate was kept constant at 0.5 ml/min and 250 µl were collected. Fractions 7–28 were analyzed by immunoblot. Peak fractions of PINK1 were further profiled by loading fractions 8–18 separately. 10 µl of each fraction were used for the analysis.

### Cell lysis, gel electrophoresis, and immunoblots

For whole cell lysates, cells were lysed in RIPA buffer (50 mM Tris-Cl, pH 8.0, 150 mM NaCl, 1% NP-40, 0.5% deoxycholate [Sigma-Aldrich, D6750], 0.1% SDS) with addition of protease and phosphatase inhibitors. Protein concentration was determined by bicinchoninic acid (Thermo Fisher Scientific, 23225). For phosphatase treatment, lysates were prepared in RIPA buffer without phosphatase inhibitors and then incubated in the absence or presence of FastAP thermosensitive alkaline phosphatase (1 U/µg protein; Thermo Fisher Scientific, EF0651) in 1x enzyme buffer for 24 h at 37°C.

Samples were mixed with 6x SDS-PAGE sample buffer, incubated for 5 min at 95°C and loaded on 8–16% Tris-Glycine gels (Thermo Fisher Scientific, EC60485BOX). Proteins were transferred onto polyvinylidene fluoride (PVDF) membranes (Millipore, IEVH00005). For experiments with monomeric Ub a 0.2-µm nitrocellulose membrane (Bio-Rad, 1620112) was used. Membranes were blocked in 5% skim milk in TBST (50 mM Tris-Cl, pH 7.4, 150 mM NaCl, 0.1% [v:v] Tween-20 [Sigma-Aldrich, P1379]) for 1 h at room temperature followed by overnight incubation with primary antibodies at 4°C. Membranes were incubated for one hour with HRP-conjugated secondary antibodies (1:10,000; Jackson Immunoresearch Laboratories, 715–035-150, 711–035-152, 703–035-155) in 5% milk:TBST. Bands were visualized with Immobilon Western Chemiluminescent HRP Substrate (Millipore, WBKLS0500) on Blue Devil Lite X-ray films (Genesee Scientific, 30–810 L) or by using a LAS-3000 (Fujifilm Life Science, Cambridge, MA, USA) or a Chemidoc MP system (Bio-Rad, Hercules, CA, USA).

For phos-tag gel electrophoresis, freshly prepared samples were separated on 6% (PINK1) or 14% (Ub) Tris-Glycine gels containing 50 µM Phos-tag acrylamide AAL-107 (Wako Chemicals, 304–93521) and 100 µM MnCl_2_. Prior transfer onto PVDF membranes, gels were soaked in ddH_2_O, then washed once for 20 min in transfer buffer containing 10 mM EDTA and 0.01% of SDS and twice in the absence of EDTA. Silver staining was performed according to the manufacturer’s instructions (Thermo Fisher Scientific, 24612).

### Antibodies

The following antibodies were used for immunoblot (IB) and immunofluorescence (IF) staining: mouse anti-GAPDH (IB; Meridian Life science, H86504M; 1:150,000), mouse anti-HSPD1/HSP60 (IB; Proteintech, 66041-1-lg; 1:5,000), mouse anti-LAMP2 (IF; Developmental Studies Hybridoma Bank, H4B4-c; 1:1,000), chicken anti-MAP2 (IF; Abcam, ab5392; 1:2,000), mouse anti-MFN2 (IB; Abcam, ab56889; 1:5,000), rabbit anti-PINK1 (IB; Cell Signaling Technology, 6946; 1:2,500), mouse anti-PINK1 (IB; Biolegend, 846201; 1:1,000), rabbit anti-p-S228-PINK1 (IB; Cell Signaling Technology, 46421; 1:1,000), mouse anti-PRKN (IB; Cell Signaling Technology, 4211; 1:1,000, for *in vitro* and overexpression experiments 1:50,000), mouse anti-PRKN (IB; Millipore, MAB5521; 1:5,000), rabbit anti-RAB8A (IB; Abcam, ab241061; 1:2,000), rabbit anti-p-S111-RAB8A (IB; Abcam, ab267492; 1:1,000), rabbit anti-MAPT/Tau (IB, E1; provided by Dr. Leonard Petrucelli [Mayo Clinic, Jacksonville, FL, USA] [50]; 1:1,000), rabbit anti-TH/tyrosine hydroxylase (IB; Millipore, AB512; 1:1,000), mouse anti-TOMM20 (IF; Santa Cruz Biotechnology, sc-17764; 1:2,000), rabbit anti-TOMM20 (IF; Proteintech, 11802-1-AP;1:250), rabbit anti-TOMM70 (IB; Proteintech, 14528-1-AP; 1:5,000), chicken anti-TUBB3/bIII-tubulin (TUJ, IB; Millipore, AB9354; 1:1,000), rabbit anti-p-S65-Ub (IB/IF; custom made [51]; 1:10,000/1:250), rabbit anti-p-S65-Ub (IB; Cell Signaling Technology, 62802; 1:20,000), mouse anti-VDAC1 (IB; Abcam, ab14734; 1:5,000), mouse anti-VCL/vinculin (IB; Sigma-Aldrich, V9131; 1:500,000). The generation and validation of the custom-made polyclonal rabbit p-S65-Ub antibody has been previously described in detail [51]. Tagged proteins were detected by streptavidin-HRP (Thermo Fisher Scientific, 21130; 1:100,000), mouse anti-V5 (IF; Thermo Fisher Scientific, R960-25; 1:300), rabbit anti-V5 (IB; Abcam, ab9115; 1:5,000), mouse anti-FLAG-HRP (IB, M2; Sigma-Aldrich, A8592, 1:20,000), mouse anti-His (IB, Takara Bio; 631212;1:1,000).

### Immunoprecipitation, kinase assays and LC-MS/MS analysis of (phospho-)PINK1

For PINK1-V5 and endogenous RAB8A immunoprecipitation (IP), cells were lysed co-IP buffer (50 mM HEPES-NaOH, pH 7.5, 10 mM KCl, 150 mM NaCl, 1 mM EDTA, 0.5 mM EGTA, 1.5 mM MgCl_2_, 10% glycerol, 0.2% NP-40) that was supplemented with protease and phosphatase inhibitors and briefly sonicated. Total cell lysate was incubated overnight at 4°C with mouse anti-V5-agarose beads (Sigma-Aldrich, A7345). RAB8A was immunoprecipitated with addition of 1 µl RAB8A antibody (Abcam, ab241061) and agarose A beads (Millipore, 16–156) were added the next day for 4 h. Formed immuno-complexes were spun down at 1000 x g, for 1 min, washed three times with co-IP buffer and eluted from beads with 2x SDS-PAGE sample buffer for 5 min at 95°C.

For kinase assays, formed immuno-complexes were spun down, washed 2x with co-IP buffer and once with *in vitro* phosphorylation buffer (20 mM HEPES, pH 7.4, 50 mM NaCl, 5 mM MgCl_2_, 0.1 mM EGTA) with addition of 0.01% Triton X-100 (Sigma-Aldrich, X100). Beads were then incubated in 100 µl of buffer supplemented with 1 µg of N-terminally biotinylated Ub (Boston Biochem, UB-560-050), 1 mM TCEP (Goldbio, TCEP1), 2 mM ATP (Sigma-Aldrich, A1348), 0.01% Triton X-100 at 37°C for 24 h.

For PINK1 phosphorylation analyses, samples were separated by SDS-PAGE and stained with Coomassie Brilliant Blue. Bands around 60 kDa were cut out, destained in 50% acetonitrile, 50 mM Tris, pH 8.2 until clear, and then reduced with 25 mM TCEP, 50 mM Tris, pH 8.2 at 60°C for 30 min, followed with alkylation using 10 mM iodoacetamide:50 mM Tris pH 8.2 at room temperature for 30 min in the dark. Proteins were digested in-situ with 0.1 ug trypsin (Promega, VA9000) in 25 mM Tris, pH 8.2, 0.0002% Zwittergent 3–16 (Calbiochem, 693023), at 37°C overnight, followed by peptide extraction with 2% trifluoroacetic acid and acetonitrile. The extracted peptides were analyzed using a Thermo Scientific Eclipse Tribrid mass spectrometer coupled to a Vanguish Neo HPLC system. The digest mixtures were loaded onto a Halo C18 2.7 µm EXP stem trap (Optimize Technologies) and chromatography was performed using 0.1% formic acid in both the A solvent (98% water:2% acetonitrile) and B solvent (80% acetonitrile:10% isopropanol:10% water), with a 3% B to 40% B gradient over 60 min at 350 nl/min through a PepSep C18 2.4 um, 100 um x 40 cm column (Bruker, 1895837). The Eclipse mass spectrometer was set for data dependent acquisition with a 2 second cycle time between the MS1 survey scan from 340–1500 m/z at resolution 120,000 (at 200 m/z), followed by EThcD MS/MS scans at resolution 30,000 with the isolation width set to 1.6 m/z. Ions with charge states of 2 to 4 were allowed for MSMS and, if selected, were placed on an exclusion list for 60s. The normalized AGC target settings were 200% for the MS1 and 200% for MS2 scans with max ion inject times of 50 ms and 54 ms, respectively.

The raw files were processed with Proteome Discoverer (ver. 2.5.0.400), searching with SequestHT against a Swissprot human database that contained the PINK1 variants (2021_01; 8135 entries) with static modifications set for carbamidomethyl cysteine. The variable modifications allowed were phosphorylation at serine, threonine, and tyrosine, and oxidation at methionine. Peptide identifications were filtered at 1% false discovery rate and phospho-site confidence required a probability of 75% or higher.

### Analysis of PRKN Ub charging

HeLa 3xFLAG-PRKN^C431S^ control cells and cells transfected with *PINK1* siRNA and siRNA-resistant *PINK1* were treated with 10 µM CCCP were harvested in preheated (95°C) SDS lysis buffer (50 mM Tris, pH 7.6, 150 mM NaCl, 1% SDS). Lysates were homogenized by 10 strokes with a 20 G needle. To verify the band shift by oxyester formation, aliquots of lysates were treated with or without NaOH (final concentration 100 mM) for 1 h at 37°C before loading onto SDS gels. 3 µg protein were analyzed by IB.

### High-content imaging (HCI) and immunostaining of cells

HeLa or HeLa EGFP-PRKN cells transfected with *PINK1* siRNA and siRNA-resistant *PINK1* cDNA were seeded with 5,000 cells per well in 96-well imaging plates (Fisher Scientific, 08772225). Twenty-four h after plating, cells were treated with 10 µM CCCP, fixed for 10 min with 4% PFA and stained with Hoechst 33,342 (1:5,000: Thermo Fisher Scientific, H21492). HeLa cells were incubated with primary anti-p-S65-Ub and anti-V5 antibodies for 2 h, followed by incubation with secondary antibodies (anti-rabbit IgG Alexa Fluor 488 and anti-mouse IgG Alexa Fluor 568; Thermo Fisher Scientific, A11034, A11004). HCI plates were imaged on a BD Pathway 855 system (BD Biosciences, Franklin Lakes, NJ, USA) with a 20x objective using a 3 × 3 montage (no gaps) with laser autofocus every second frame. Raw images were processed using the built-in AttoVision V1.6 software. Regions of interest (ROIs) were defined as “cytoplasm” and “nucleus” using the built-in “RING 2 outputs” segmentation for the Hoechst channel after applying a shading algorithm [28]. For p-S65-Ub, the cytoplasmic intensity of each cell was normalized to the respective V5 intensity. EGFP-PRKN translocation measurements [28] were restricted to mCherry-positive cells, defined as cells with mCherry signal equal or higher the overall average intensity. ReN cells VM were grown on Matrigel coated coverslips and differentiated. Cells were fixed with 4% paraformaldehyde for 10 minutes before membranes were permeabilized with 1% Triton X-100 in PBS (Boston Bioproducts, BM-220) for 10 min. Cells were stained with TOMM20, LAMP2, and MAP2 antibodies, followed by secondary antibodies (anti-rabbit IgG Alexa Fluor 488, anti-mouse IgG Alexa Fluor 568, anti-chicken Alexa Fluor 647, Thermo Fisher Scientific, A11034, A11004, A21449). Nuclei were counterstained with Hoechst 33342 (1:5000 in PBS). Representative images were obtained by AxioObserver microscope equipped with an ApoTome Imaging System (Zeiss, Oberkochen, Germany).

### CRISPR-Cas9 gene-editing and lentivirus

We used CRISPR-Cas9 to introduce a single nucleotide exchange by replacing the GGC (Gly411) with an AGC (Ser411) or a GCC (Ala411) triplet. HEK293T cells were transfected with 1 µg pX330-sgRNA-Cas9 plasmid (Addgene, 42230; deposited by Feng Zhang) and 10 μg single stranded oligo (Integrated DNA Technologies) using X-tremeGENE 9 (Sigma-Aldrich, 6365787001) according to the manufacturer’s instructions. ReN cells VM stably expressing mitoKeima were transfected using the nucleofector P3 kit (Lonza, V4XP-3032). Single cell colonies were generated by limited dilution of transfected cells in 96-well plates. All clones were analyzed by Sanger sequencing. Sequences were analyzed using SnapGene software. In the case of ReN cell VM, we introduced a silent blocking mutation downstream of the intended change that allowed prescreening of clones with a BanI restriction digest. Because of a triplication of chromosome one in HEK293T cells, we further amplified genomic DNA of positive candidates with PCR and cloned the PCR products into a pGEM T Easy vector (Promega, A1360) to confirm that all alleles had been edited correctly. After confirming on-target editing, we also excluded off-targets by Sanger sequencing of candidate regions as identified by the Benchling biology software (2021, www.benchling.com) and confirmed similar expression of *PINK1* and *PRKN* by qRT-PCR and IB. For HEK293T cells, we further monitored cellular ROS (CellRoX), mitochondrial ROS (mitoSOX), mitochondrial membrane potential (JC10), and cellular respiration (Seahorse mitochondrial stress test; Agilent, 103015–100) and found no difference to parental, un-edited cells. Further modification of genome-edited HEK293T cells was performed using lentivirus to knock out *PRKN* with a sgRNA targeting the start codon of PRKN. Antibiotics were used to select positive cell populations and single clones were analyzed by immunoblot. HEK293E *PINK1* KO cells have been described previously [30].

### Meso Scale Discovery ELISA

96-well Meso Scale Discovery assay plates (Meso Scale Diagnostics, L15XA-3) were coated with p-S65-Ub (Cell Signaling Technology, 62802) capturing antibody with a final concentration of 1 µg/ml in carbonate-bicarbonate coating buffer (15 mM Na_2_CO_3_, 35 mM NaHCO_3_, pH 9.4) overnight at 4°C. Plates were washed three times with 0.2% Tween-20 in TBS (TBST), blocked in 5% BSA in TBST for 1 h at room temperature and washed again. Protein lysates were prepared in NP40 lysis buffer (150 mM NaCl, 1.0% NP-40, 50 mM Tris-Cl, pH 8.0 containing protease and phosphatase inhibitors) diluted in 1% BSA in TBST to final concentration 0.5 µg/µl and 50 µl of diluted lysates was incubated overnight on the plate at 4°C. Plates were washed and incubated with mouse anti-total-Ub detection antibody (1:500, Millipore, Ubi-1, MAB1510; or 5 ug/ml, Santa Cruz Biotechnology, P4D1, sc8017) for 1 h, followed by three washes and incubation with goat anti-mouse SULFO TAG secondary antibody (1:500 in 1% BSA in TBST; Meso Scale Diagnostics, R32AC-1) for 1 h at room temperature. Plates were washed and signal measured after addition of 150 μl/well 2x read buffer (Meso Scale Diagnostics, R92TG-2) with surfactant on a SECTOR Imager 2400 using the Meso Scale Discovery workbench Software as recently described [30].

### Quantitative mass spectrometry of mitochondria

HEK293T cells expressing WT PINK1, or CRISPR edited to express PINK1^G411A^ or PINK1^G411S^ were treated with 10 µM CCCP for the time indicated. Cells were harvested and subjected to crude mitochondrial isolation as described previously [20,31]. Briefly, crude mitochondria samples were prepared by sonication of cells resuspended in mitochondrial isolation buffer (50 mM Tris-HCl, pH 7.5, 70 mM sucrose [Sigma-Aldrich, S9378], 210 mM sorbitol [Sigma-Aldrich, S1876], 1 mM EDTA, 1 mM EGTA, 50 mM NaF, 5 mM sodium pyrophosphate, 10 mM sodium 2-glycerophosphate [Calbiochem, 35,675], 1 mM AEBSF [Sigma-Aldrich,76,307], 10 mM PR-619 [Selleck Chemicals, S7130], 1 mM benzamidine [Sigma-Aldrich, B6506], 1 mg/ml leupeptin [Sigma-Aldrich, L2023] and aprotinin [Roche, 10,236,624,001]) plus 100 mM chloroacetamide (Sigma-Aldrich, C0267) and mitochondria were then isolated by differential centrifugation before lysis. Mitochondrial extracts were sonicated, clarified by centrifugation, and protein concentrations determined by the Bradford assay (Bio-Rad, 5,000,006). Quantitative mass spectrometry was performed on mitochondrial extracts (3 µg) using PRKN target-Parallel Reaction Monitoring (Pt-PRM) platform using an optimized series of heavy reference peptides for PRM analysis of Ub chain linkages, and p-S65-Ub [52] and data were collected on an Orbitrap Fusion Lumos instrument.

### Expression and Purification of Ub-WT-6XHistidin and Ub-TVLN-6XHistidin

Human Ub DNA was subcloned into a pET60 vector (Novagen, 71851) for ubiquitin-6His-tag. PCR mutagenesis was used to generate Ub[TVLN]-6His-tag mutant. The corresponding proteins were produced in Rossetta (DE3) E. coli cells grown in 1 L terrific broth (Sigma-Aldrich, T0918). When the OD reached 0.6, the proteins were induced using 1 mM IPTG (Goldbio, I2481C) and further incubated for 21 h at 25°C. The E. coli cells were then collected, resuspended in 40 ml lysis buffer (50 mM Tris-HCl, pH 7.5, 150 mM NaCl, 1% Triton X-100, 0.06% BME, 2 tablets of Roche protease inhibitors), and lysed by tip-sonication. After removing the cell debris through ultracentrifugation (20,000 x g, 10 min), the supernatant was filtered through a 0.2-µm filter and incubated with the Ni-NTA beads (Pierce, 88221). After three times of wash, the 6XHis tagged proteins were eluted with Tris-HCl buffer containing 500 mM imidazole. The eluate was then loaded on a gel-filtration Superose 6 10/300 GL (GE Healthcare, 17517201) for further purification. Purity and integrity of the proteins were confirmed by Coomassie Brilliant Blue staining and intact mass analysis using Agilent 6130 Single Quadrupole Mass Spectrometer with 1260 LCMS (Expected MS for Ub^WT^-6xHis (max isotopic m/z) = 9387.99127, measured [M + H] = 9388.03; Expected MS for Ub^TVLN^-6xHis (max isotopic m/z) = 9386.97084, measured [M + H] = 9387.03).

### Mitochondrial preparations and kinase assays with mitochondria

For fractionation into mitochondrial and cytoplasmic fraction cells were washed scraped in fractionation buffer (20 mM HEPES, pH 7.6, 220 mM mannitol, 10 mM potassium acetate, 70 mM sucrose) containing protease and phosphatase inhibitor cocktails and homogenized with 10 strokes through a 27 G needle. Lysates were spun at 800 x g for 5 min at 4°C to remove nuclei and post-nuclear supernatant was spun at 8000 x g for 20 min at 4°C to pellet mitochondria. Protein concentration was determined using a BCA assay and 50 µg of mitochondria were used per kinase assay reactions. Mitochondria were resuspended in 10 µl of kinase reaction containing 10% ATP regeneration buffer (20 mM HEPES, pH 7.6, 10 mM ATP, 300 mM phosphocreatine [Sigma-Aldrich, P7936], 10 mM MgCl_2_, 10% glycerol, 1.5 mg/ml creatine phosphokinase [Sigma-Aldrich, C3755]), 1 µg of FLAG- or His-tagged Ub (Boston Biochem, U-120, U-530) in fractionation buffer and incubated at 30°C for 2 or 4 h. Mitochondria were spun down for 5 min at 20,000 x g and supernatant was collected and mixed with 6x sample buffer; 10 µl were loaded per lane.

### Flow cytometry analysis of mitoKeima

Analysis of mtKeima was performed using an Attune NxT (B/R/Y/V) flow cytometer (Thermo Fisher Scientific, Waltham, MA, USA). Neutral mtKeima signal was collected with 405 nm excitation and 603/48 nm emission (standard VL3 detector optical path). Acidic mtKeima signal was collected with 561 nm excitation and custom optical settings for the YL2 detector, generated by replacing the 650DLP with the 740DLP filter and removing the BP620/15 filter, resulting in capture of 600–740 nm emission. Height signals were collected and analyzed in log scale, without compensation. Data were analyzed with FCS Express 6 (De Novo Software) using standard doublet discrimination, followed by viability determination with Sytox Red. The resulting single, live cells (at least 20,000 per experiment) were displayed on a bivariate plot of neutral (x-axis) vs. acidic (y-axis) mtKeima signal and the data exported for calculating the ratio of acidic to neutral Keima signal for each cell.

### Densitometry and statistical analysis

Densitometric analysis of western blots was performed by using ImageStudio Lite software version 5.2.5. Data analysis and visualization were performed by GraphPad Prism version 8. Statistical comparisons were performed using parametric analysis with either using student’s two-sided t-test, one-way and two-way ANOVA with Tukey’s post-hoc test (*p < 0.05, **p < 0.005, ***p < 0.0005), depending on the number of groups and experimental design.

### PINK1 model construction

The sequence of human PINK1 was taken from the NCBI Reference Sequence NG_008164.1. Amino acid (aa) residues 3–581 were used to generate a complete structural model. The PINK1 sequence was constructed based on multiple alignments, in which each domain was modeled as a separate unit, which were then assembled into a final composite hybrid model. The model was generated from consensus between the programs PRIME (Prime v3.0, Schrodinger, LLC, New York, NY) [53,54], and YASARA SSP/Homology/PSSM Method [55–58]. The variable loops and gaps were filled using homology and knowledge-based potentials with YASARA. Each missing loop was modeled using the Loop Search module in Sybyl 8.0 or with YASARA loop modeler [55–62]. The selection of final loops was based on highest homology, as well as lowest root mean square deviations (RMSDs). The side chains and rotamers were adjusted with empirical potentials, simulated annealing with explicit solvent, and small equilibration simulations using YASARA’s refinement protocol. These were verified by WHAT-IF and PROCHECK [63].

Refinement of the hybrid model was carried out using a limited molecular dynamics-based refinement in YASARA consisting of the Secondary Structure Prediction (SSP) for YASARA parameterization, pKa assignment, solvation and simulated annealing and pre-equilibration setup, i.e. energy minimizations [55,56,59,61]. Both homology and fold recognition were considered, and a final refinement with the entire model was completed using YASARA for 250 psec of molecular dynamics embedded in an empirical force field. The model was then subjected to energy optimization with Polak-Ribiere conjugate gradient (PRCG) with an R-dependent dielectric for finalization.

### Molecular dynamics simulation (MDS)

Molecular dynamics was completed on each model for conformational sampling in order to examine any dynamical conformational differences that may occur between the WT or G411A variant. Briefly, each PINK1-Ub system was minimized with relaxed restraints, using steepest descent (SD) PRCG, and equilibrated in solvent with physiological ionic strength [64–67]. The refinement included the following steps: (1) Energy minimization of explicit water molecules and ions, with protein constrained; (2) Energy minimization of the entire system; (3) MDS for >10 nsec to ensure system acclimatization to the force field (OPLS3/Amber) [68,69]. Succeeding the refinement protocol, 1.5 µsec simulations were completed to collect data for analyses. More complete detail of our minimization/equilibration refinement protocol, as well as simulation can be found in the literature [70–73].

### MDS and modeling analytical methods

Clustering was performed using the Quality Threshold algorithm [74,75] with RMSD cutoff of 1.75 Å (determined heuristically) for both PINK1 WT and G411A. The central structure for each cluster was uploaded to PyMOL and Ub-CR (1ubq.pdb) [76] or Ub-common (6eqi.pdb) [23] crystal structures were aligned to each of them in order to obtain RMSD. FoldX plugin with YASARA [77,78] was used to calculate the dG interaction energy between ubiquitin and PINK1 for each central cluster member, which was then multiplied by the percent of simulation time that conformation was adopted and to generate a weighted dG interaction score to account for conformational propensity.

Post-MDS analyses were performed using VMD [79]. The random rotation/translation was removed via alignment (least squares CONCa backbone superposition onto first frame) as specified in each figure, e.g., ubiquitin chain. RMSD over time was calculated using VMD’s RMSD trajectory tool. RMSF was calculated using a custom Tcl/Tk script. Secondary structure per residue per frame was calculated via VMD’s Timeline plugin, followed by a custom Visual Basic function to calculate the percent occupancy of specific secondary structure characters. SASA was calculated via VMD’s Timeline plugin. Ramachandran distribution plots were generated via VMD’s Ramaplot plugin. Hydrogen bonds measurements were taken using the Hydrogen Bonds analysis extension in VMD. The BSA for each frame was computed via a custom Python script measuring and summing the solvent accessible surface area (SASA) of each chain separately, then subtracting the SASA of the complex, then dividing by 2 for correction (SASA PINK1 + SASA Ub – SASA complex)/2, where the SASA probe radius is 1.4 Å).

Grcarma [80] was used to generate mass-weighted, normalized covariance matrices. These matrices were then plotted as 2D graphs with respect to residues of interest, as well as appended to the beta column via a custom script and displayed using PyMOL (The PyMOL Molecular Graphics System, Version 2.0 Schrödinger, LLC.) All molecular structure graphics were generated with PyMOL.

## Supplementary Material

Supplemental Material

## Data Availability

All data generated or analyzed during this study are included in this published article and its supplementary information files.
