# Supplementary material for: Substitution of PINK1 Gly411 modulates substrate receptivity and turnover
Source: Autophagy. 2022 Dec 5;19(6):1711–32. doi: 10.1080/15548627.2022.2151294 (PMC10262784; doi:10.1080/15548627.2022.2151294)
Supplement: Supplemental Material [file KAUP_A_2151294_SM8220.zip › PINK1 Gly411_SupplementaryInformation.pdf]

SUPPLEMENTARY INFORMATION

A

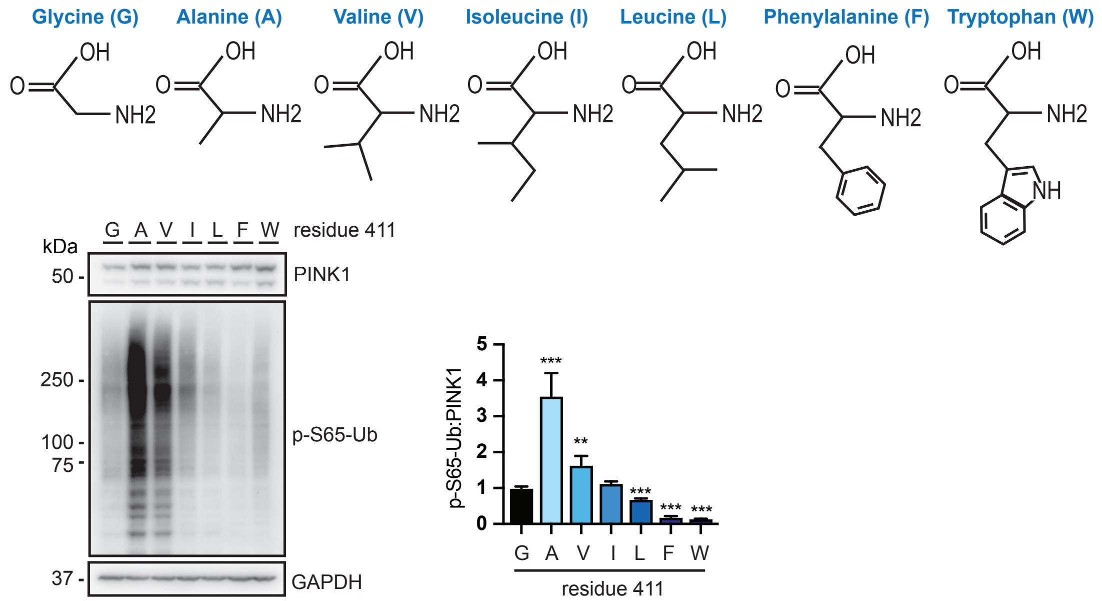

B

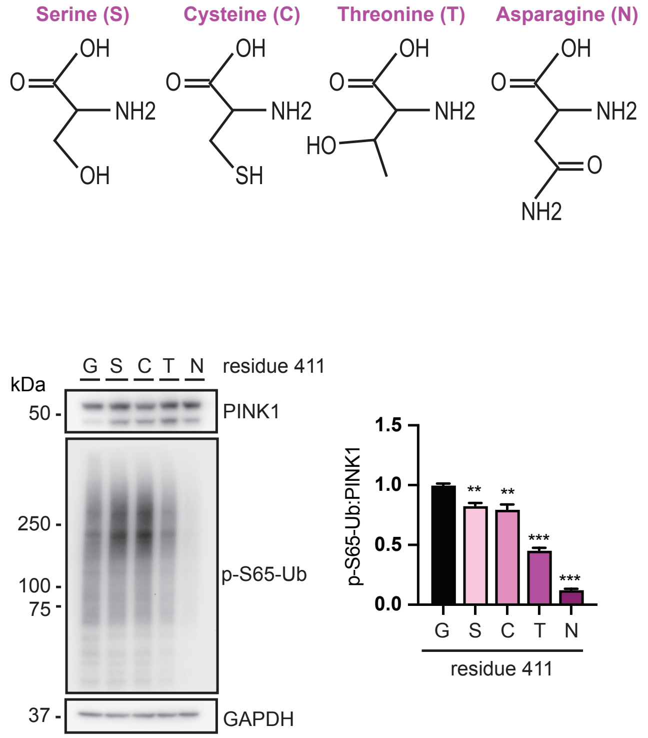

C

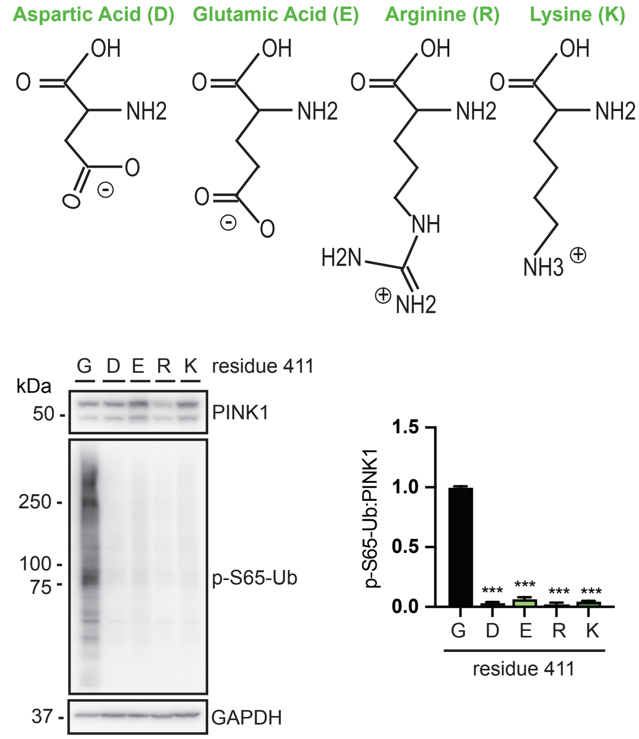

Figure S1. Short non-polar amino acids at position 411 boost PINK1 Ub kinase activity. (**A-C**) Hek293E *PINK1* KO cells were transiently transfected with PINK1-V5 constructs bearing various amino acids at position 411. Cells were treated with CCCP for 1 h and lysates analyzed by p-S65-Ub ELISA assay. Values were normalized to PINK1 levels to correct for differences in transfection levels and compared to WT PINK1 (G411). Data was grouped in three sets: non-polar (A), polar (B) and charged side chains (C). For each set, the amino acid structures are shown alongside a representative immunoblot and the quantitative data. p-S65-Ub to PINK1 levels are shown as mean +/- SEM from at least three experiments. Statistical analysis was performed by pairwise comparison to WT with a two-sided t-test (***, $p<0.0005$, **, $p<0.005$, absence of asterisks denotes $p>0.05$).

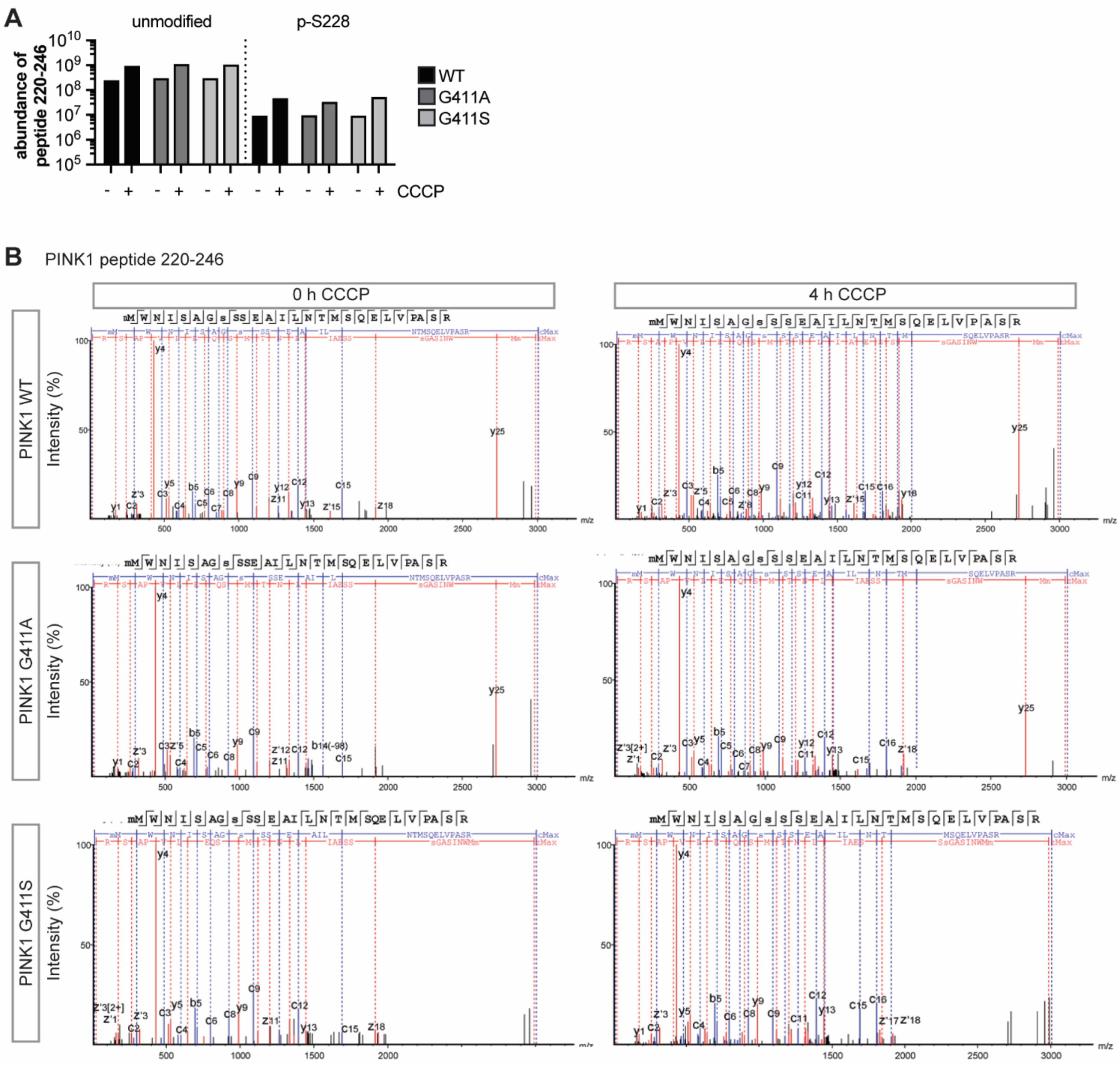

Figure S2. Mass spectrometry of PINK1-V5 indicates similar autophosphorylation at S228. (**A-B**) PINK1-V5 was immunoprecipitated from HEK293E cells and subjected to mass spectrometry. (A) Shown is the relatively abundance of unphosphorylated and S228 phosphorylated peptide 220-246. (B) Shown are spectra of peptide 220-246 for the analyzed PINK1-V5 samples from WT, PINK1^{G411A}, and PINK1^{G411S} cells with and without 4-h CCCP treatment.

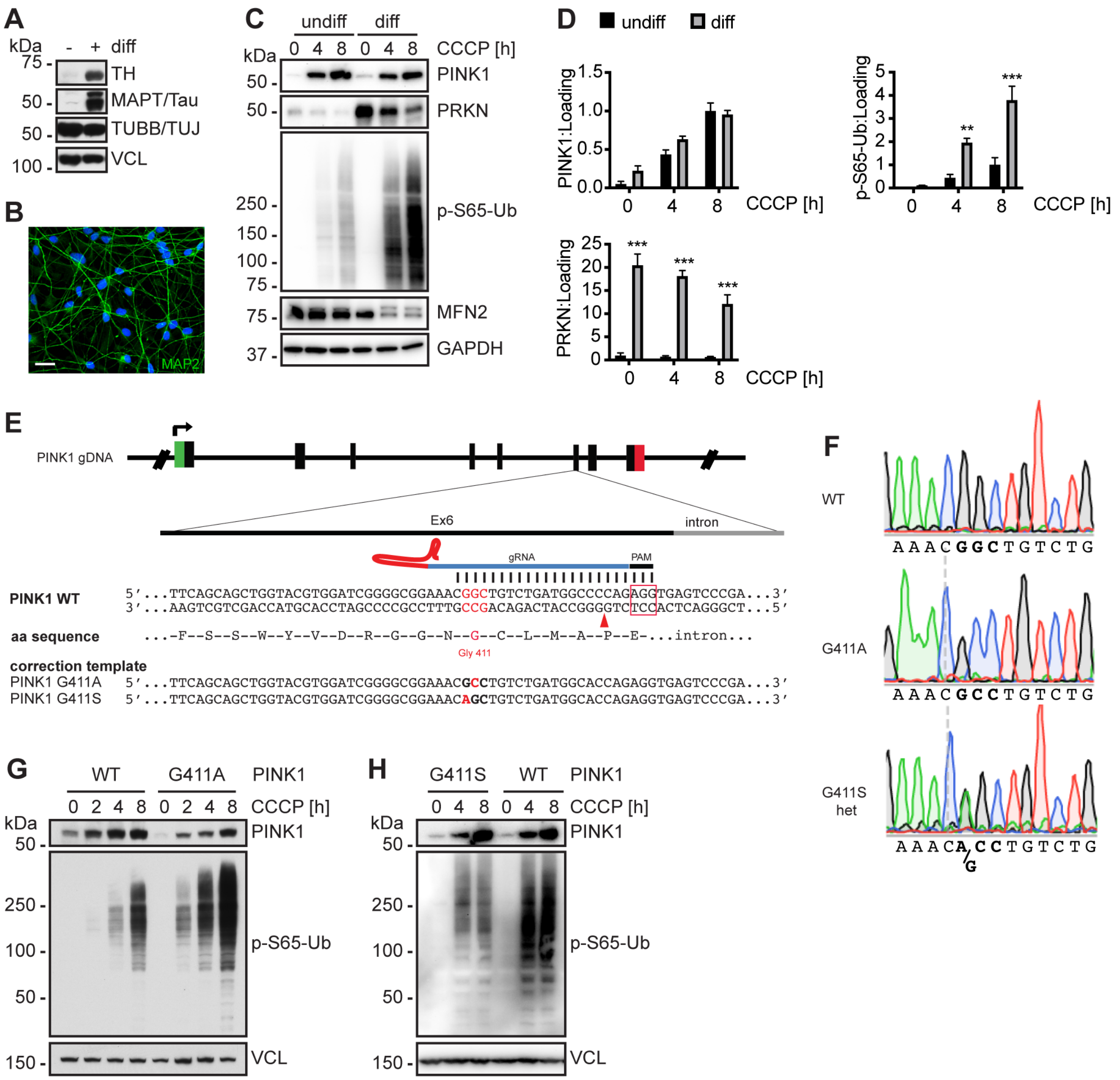

Figure S3. Generation of an isogenic neuronal cell model. **(A)** Immunoblot comparison of undifferentiated neural progenitor cells (NPCs) and differentiated TH (tyrosine hydroxylase)-positive neurons after differentiation for 7 days in growth-factor free media. TUBB3/TUJ1 served as a general neuronal marker, TH as a dopamine neuron specific marker, tau as neuronal maturation marker, and VCL as a loading control. **(B)** Immunofluorescence imaging of MAP2-stained cells shows neuronal morphology of differentiated cells. Nuclei are stained with Hoechst

(blue). Scale bars: 50 µm. **(C)** Immunoblot comparison of undifferentiated (undiff) NPCs vs. differentiated (diff) mature neurons that have been treated with CCCP for the indicated times. Representative blots are shown for PINK1, PRKN, and p-S65-Ub as well as for the PRKN substrate MFN2. GAPDH served as a loading control. **(D)** Quantification of three independent experiments as shown in C confirmed a statistically significant increase in PRKN and p-S65-Ub but unaltered levels of PINK1. **(E)** Overview over the strategy to substitute residue 411 of PINK1 with A or S in human NPCs using CRISPR-Cas9. **(F)** Sanger sequencing confirmed the successful gene editing. **(G-H)** Differentiated ReN cell VM expressing heterozygous PINK1^{G411S} (G) or undifferentiated PINK1^{G411A} (H) and parental control cells were treated with 20 µM CCCP for the indicated times. Immunoblot analysis of p-S65-Ub shows increased levels in homozygous PINK1^{G411A} knockin cells and decreased levels in heterozygous PINK1^{G411S} cells, while PINK1 levels are unaltered.

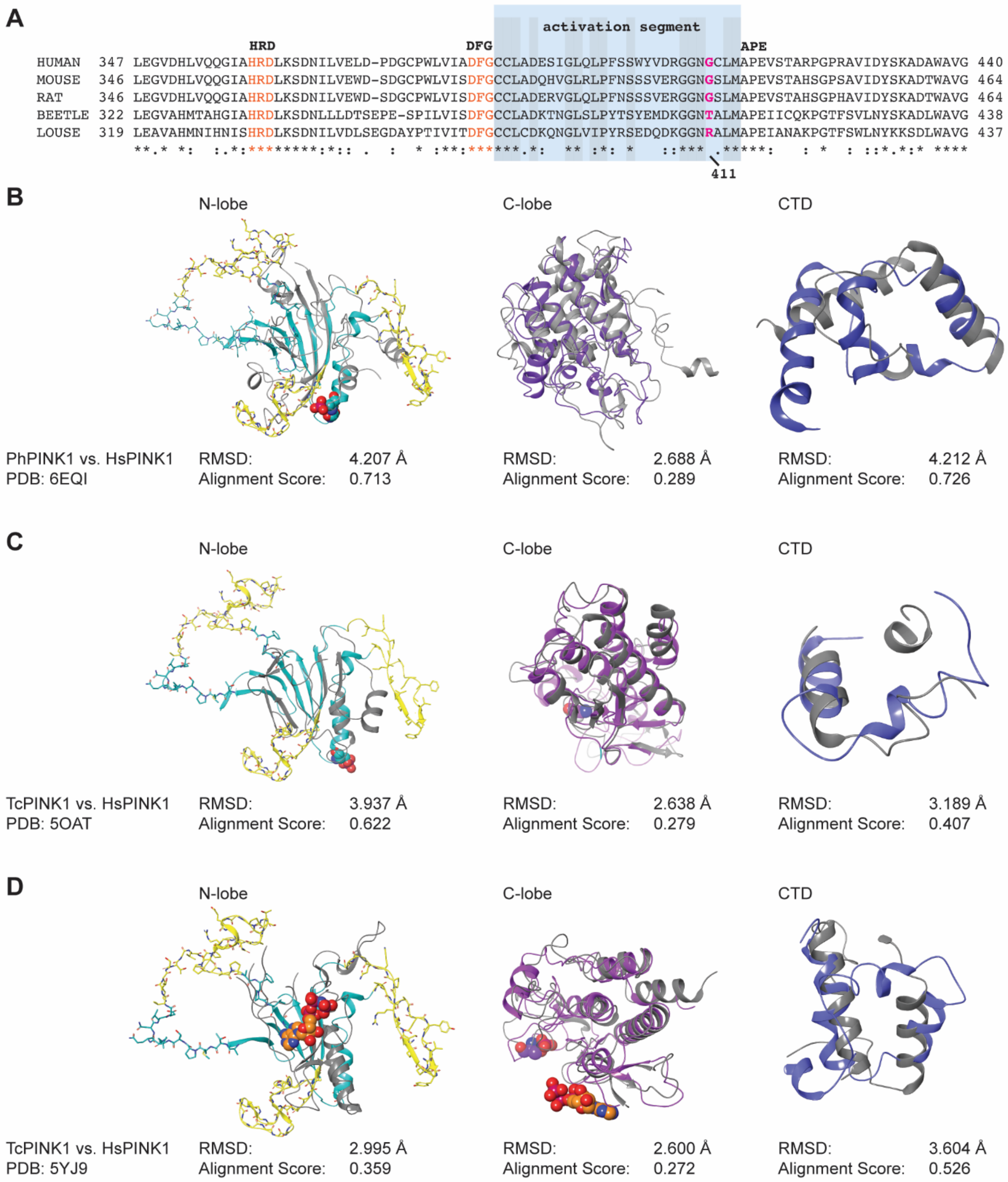

Figure S4. Domain-by-domain comparison between human PINK1 model and available crystal structure information from insect homologs. **(A)** Shown is a sequence alignment of parts of the

PINK1 kinase domain of human, mouse, rat, red flour beetle (*Tribolium castaneum*) and body louse (*Pediculus humanus* subsp. corporis) origin. Highlighted are the conserved HRD, DFG, and APE motifs as well as the activation segment that contains the residue G411. **(B-D)** Shown are superposition images of structural alignments for individual domains of PINK1 (from left to right: N-lobe, C-lobe, and CTD). Human PINK1 (HsPINK1) is color-coded by domain while the respective crystal structure fragments are overlaid and displayed in gray. RMSD values and alignment scores are given below each structure/domain comparison. Though there are homologous zones and the overall orientation is conserved, note that structural similarity is affected by differences in length or missing residues, especially for the insertion loops of the N-lobe or the CTD domain. Comparison between the human PINK1 model and **(B)** louse (*Pediculus humanus corporis*) PhPINK1 (PDB code: 6EQI) [23], **(C)** beetle (*Tribolium castaneum*) TcPINK1 (PDB code: 5OAT) [21], and **(D)** TcPINK1 (PDB code: 5YJ9) [22].

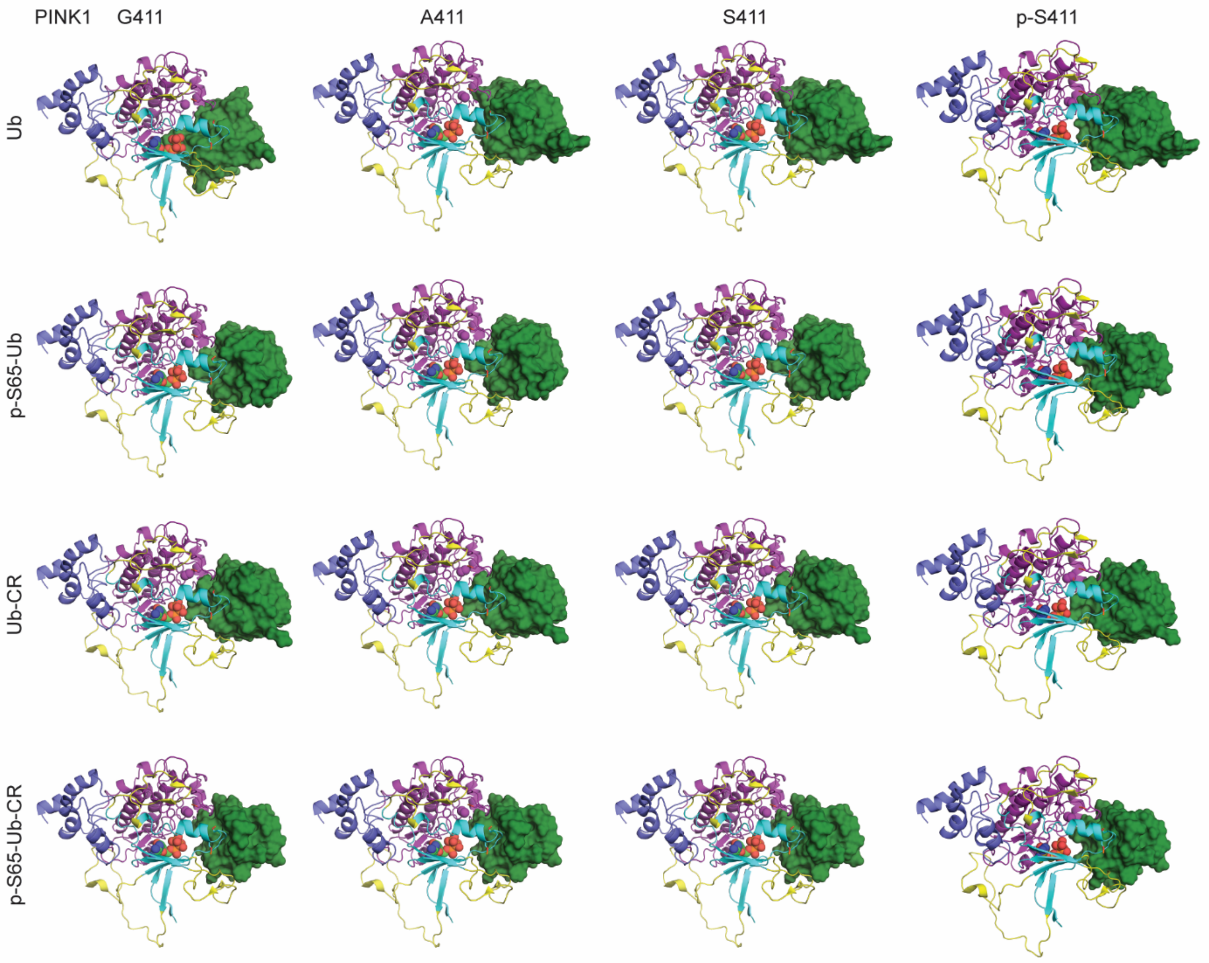

Figure S5. Overview of the modeled PINK1-Ub complexes. The 16 different substrate-enzyme complexes are presented as structurally modeled. Shown are the different PINK1 variants (from left to right: G411, A411, S411, and p-S411) docked to distinct Ub substrates (from top to bottom: Ub, p-S65-Ub, Ub-CR, and p-S65-Ub-CR). PINK1 residue 411 and the autophosphorylation site p-S228 as well as ATP and S65 of the Ub substrates are highlighted in VdW presentation.

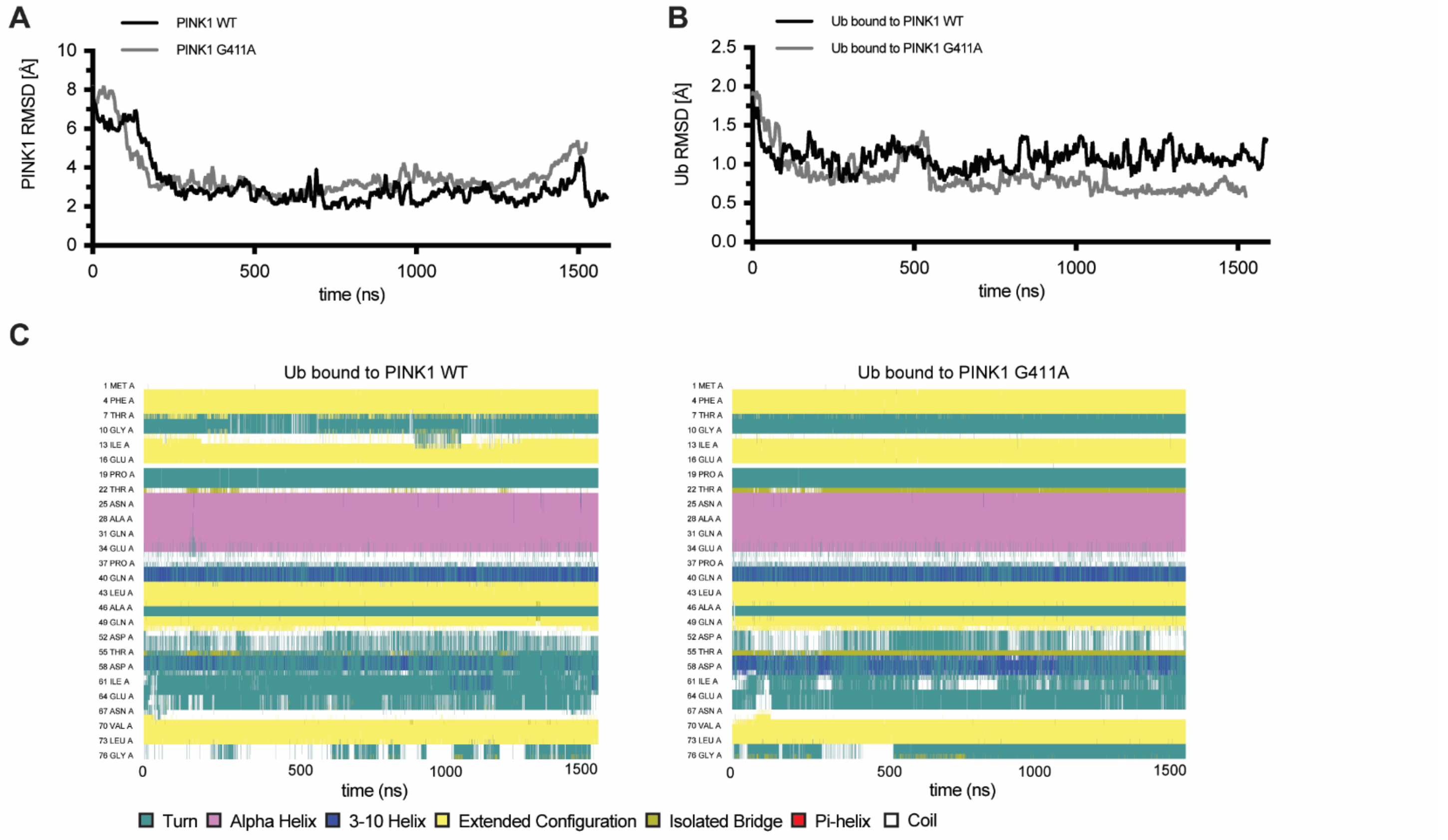

Figure S6. Structural measurements for PINK1 and Ub during molecular dynamic simulations of the enzyme-substrate complex. **(A, B)** Root mean square deviation (RMSD) shows the average distance between the atoms of the enzymatic complexes. Rotational and translational degrees of freedom were subtracted from the RMSD trajectory (using a module in VMD) to remove random whole molecule motion that occur during unbiased MDS by superposing all frames of the simulation onto the first frame as reference using only the backbone (CONCα) atoms. **(A)** RMSD of an enzymatic PINK1 fragment lacking the membrane part but containing the entire kinase domain and the CTD region (residues 156-581). **(B)** RMSD of the entire Ub substrate (residues 1-76) during the simulation in complex with WT PINK1 (black) or PINK1^{G411A} (gray). **(C)** Analysis of the secondary structure for the substrate Ub (residues 1-76) bound to WT PINK1 (left) or

PINK1^{G411A} (right) during the entire simulation. Images were generated with the Timeline module of VMD. In addition to more subtle differences seen across the entire molecule, the extreme C-terminus of Ub bound to PINK1^{G411A} showed the most pronounced changes. Quantification revealed a turn in only 35.14% of the time in case of Ub bound to WT PINK1, whereas this was increased to 79.41% in the context of PINK1^{G411A}, indicative of a more often retracted C-terminal tail of Ub.

Table S1. Calculations of free energy, proximity, and interface areas.

	Substrate		PINK1 G411	PINK1 A411	PINK1 S411	PINK1 pS411
**Energy within the enzyme-substrate complex [kcal/mol]** [a]	**Ub**	S65	0	2.24 ± 0.48	-2.82 ± 0.51	-4.09 ± 0.47
		pS65	0	-1.76 ± 0.51	-3.31 ± 0.48	-4.11 ± 0.50
	**Ub-CR**	S65	0	2.42 ± 0.42	-2.24 ± 0.51	-1.98 ± 0.32
		pS65	0	-2.97 ± 0.55	-4.05 ± 0.44	-2.77 ± 0.37
**Substrate proximity to ATP [Å]** [b]	**Ub**	S65	8.87	8.85	9.13	9.87
		pS65	14.01	13.45	13.85	14.42
	**Ub-CR**	S65	8.66	8.55	9.46	9.67
		pS65	9.77	11.57	10.47	11.09
**Ub substrate surface area [Å^2]** [c]	**Ub**	S65	4220	4166	4373	4083
		pS65	4168	4280	4224	4328
	**Ub-CR**	S65	4815	4590	4713	4637
		pS65	4544	4430	4425	4267
**PINK1 surface area [Å^2]** [c]	**Ub**	S65	31945	32004	32165	31847
		pS65	31290	31546	31424	31543
	**Ub-CR**	S65	34211	34207	34082	34156
		pS65	32024	31817	31911	31570
**PINK1-Ub interface area [Å^2]** [c]	**Ub**	S65	1068	1029.2	957.5	1007.1
		pS65	1250.1	1308.8	1249.8	1170.3
	**Ub-CR**	S65	874.3	704.8	795.6	860.5
		pS65	1185.6	1235.4	1066.2	1232.9

[a] FEP+ calculation (Gibbs free energy) for prediction of (1) binding energy of substrate to enzyme and (2) protein stability of the PINK1 variant.
[b] Distances were measured between the oxygen atom of Ser65 and the terminal ATP phosphate.
[c] PISA calculations of buried surface areas and interaction interfaces.

Table S2. Cluster energetics.

Ub bound to PINK1 WT cluster energetics							
cluster ID	% of frames	FoldX dG interaction between PINK1 and Ub (kcal/mol)	**weighted sum of dG (kcal/mol)**	complex stability (kcal/mol)	weighted sum of stability (kcal/mol)	total potential energy (kcal/mol)	weighted sum of potential energy
1	43.62%	2.07	**0.90**	649.66	283.39	1118.63	487.96
2	24.13%	0.38	**0.09**	648.57	156.51	1152.42	278.09
3	7.69%	4.23	**0.33**	650.03	50.00	1147.13	88.24
4	4.33%	0.80	**0.03**	681.12	29.46	1186.14	51.31
5	3.92%	4.90	**0.19**	672.85	26.36	1043.29	40.87
6	3.07%	7.45	**0.23**	643.31	19.73	1092.16	33.50
7	2.63%	2.12	**0.06**	635.55	16.70	1040.65	27.34
8	2.39%	-1.11	**-0.03**	636.24	15.21	1131.49	27.05
9	1.98%	2.90	**0.06**	667.86	13.24	1140.50	22.61
10	1.42%	1.88	**0.03**	661.39	9.36	1181.67	16.73
11	1.31%	4.82	**0.06**	692.23	9.04	1112.31	14.52
rejected	3.49%						
**Average or Sum**		**2.77**	**1.95**	**658.07**	**629.00**	**1122.40**	**1088.22**
StDev			**0.86**	20.13		26.73	

Ub bound to PINK1 G411A cluster energetics							
cluster ID	% of frames	FoldX dG interaction between PINK1 and Ub (kcal/mol)	**weighted sum of dG (kcal/mol)**	complex stability (kcal/mol)	weighted sum of stability (kcal/mol)	total potential energy (kcal/mol)	weighted sum of potential energy
1	86.90%	-0.98	**-0.85**	658.41	572.15	1196.01	1039.33
2	4.80%	-0.01	**0.00**	697.80	33.52	1273.40	61.17
3	4.75%	4.67	**0.22**	617.42	29.36	1062.82	50.54
4	1.71%	4.41	**0.08**	630.87	10.76	1167.49	19.91
rejected	1.80%						
**Average or Sum**		**2.02**	**-0.55**	**651.13**	**645.79**	**1174.93**	**1170.95**
StDev			**0.46**	30.73		75.44	

The center frame for each unique conformational cluster was subjected to energetics calculations using FoldX. The raw score for dG of interaction between PINK1 and Ub, complex stability, and total potential energy was then weighted by the percentage of frames which that cluster represents to account for the conformational occupancy and dynamics.
