# Supplementary figures and images for: Substitution of PINK1 Gly411 modulates substrate receptivity and turnover
Source: Autophagy. 2022 Dec 5;19(6):1711–32. doi: 10.1080/15548627.2022.2151294 (PMC10262784; doi:10.1080/15548627.2022.2151294)

# SUPPLEMENTARY FIGURE S6

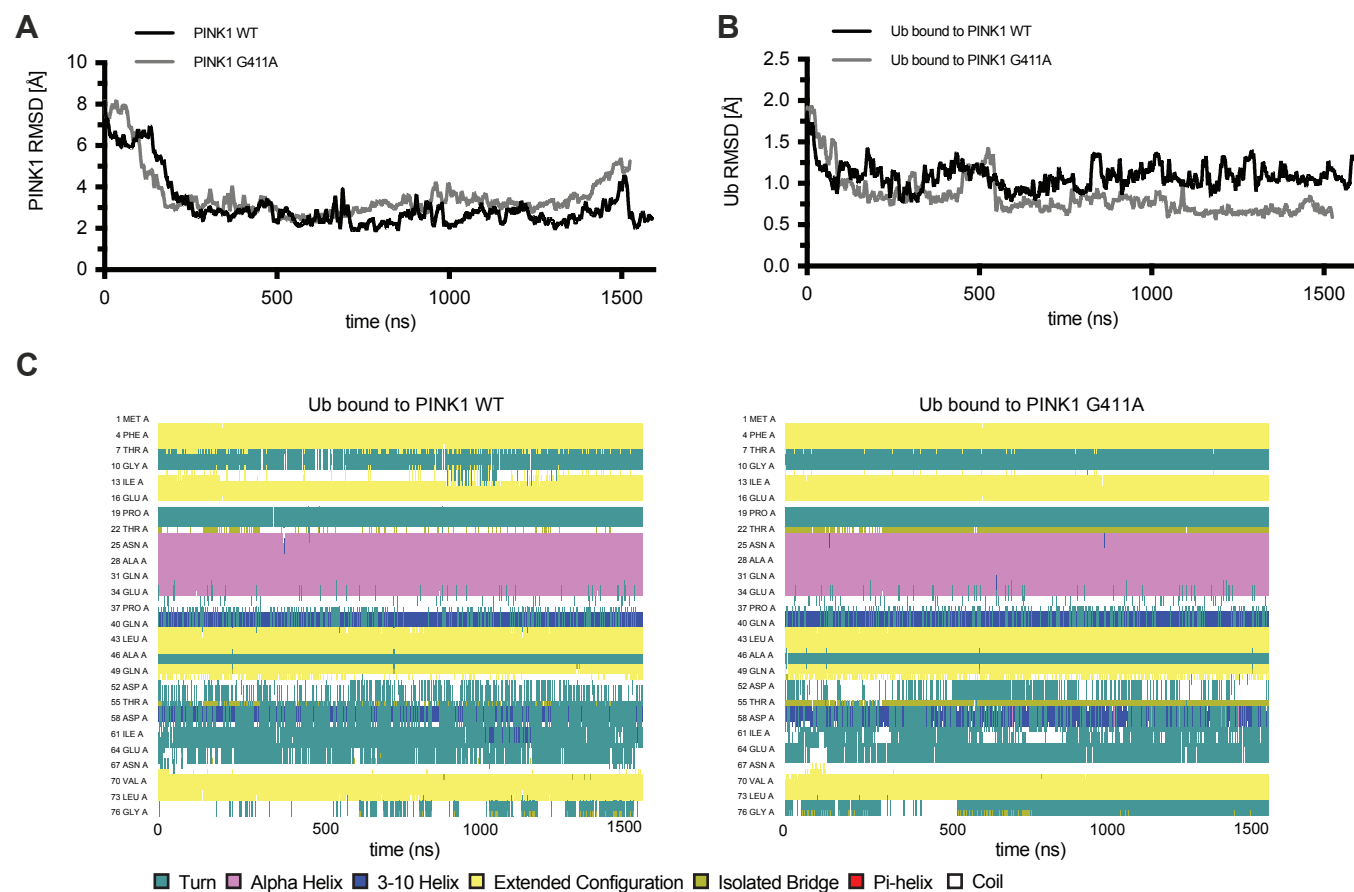

Supplement: Supplemental Material [file KAUP_A_2151294_SM8220.zip › PINK1Gly411_FigureS6.pdf]

**SUPPLEMENTARY FIGURE S5**

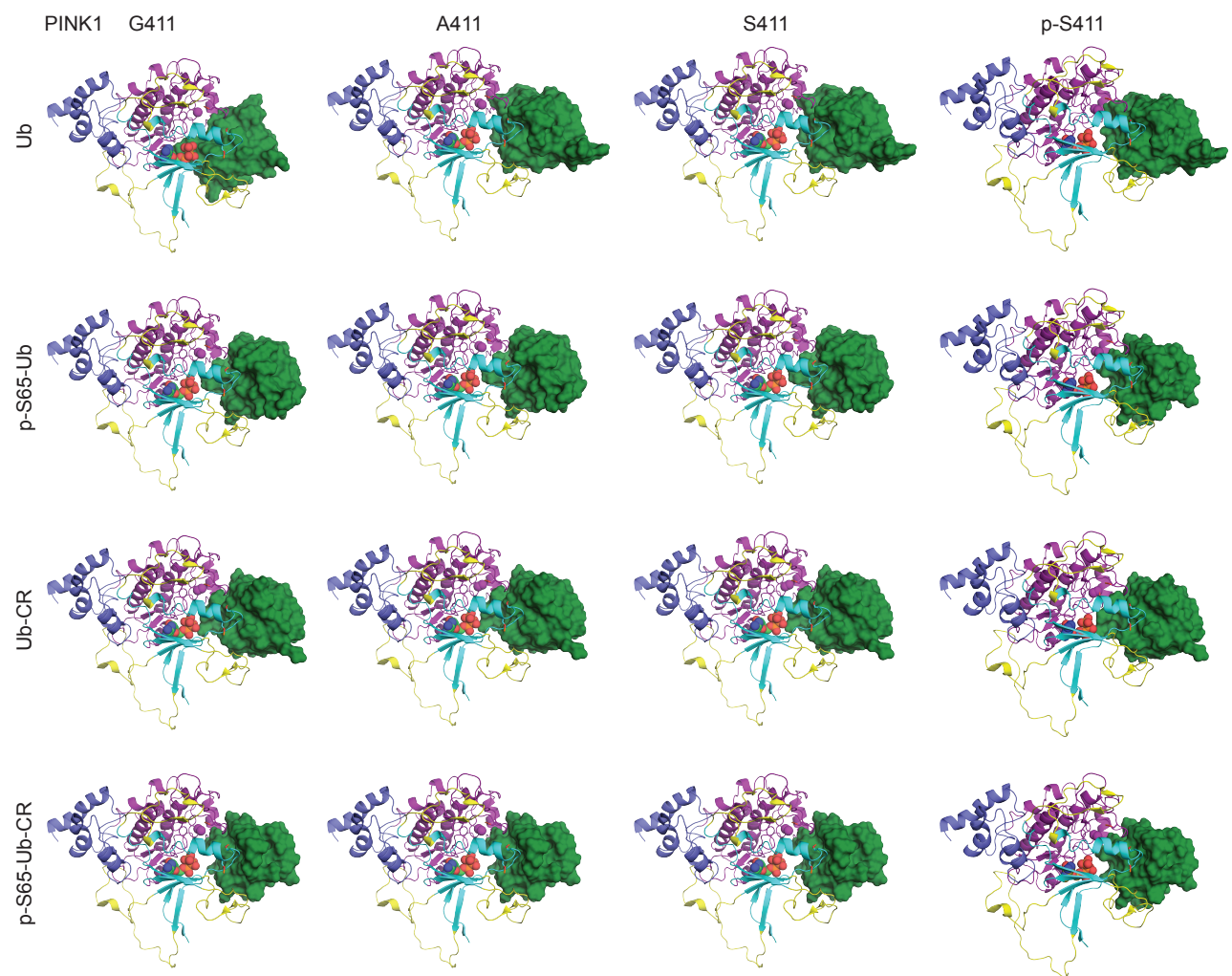

Supplement: Supplemental Material [file KAUP_A_2151294_SM8220.zip › PINK1Gly411_FigureS5.pdf]

[illegible]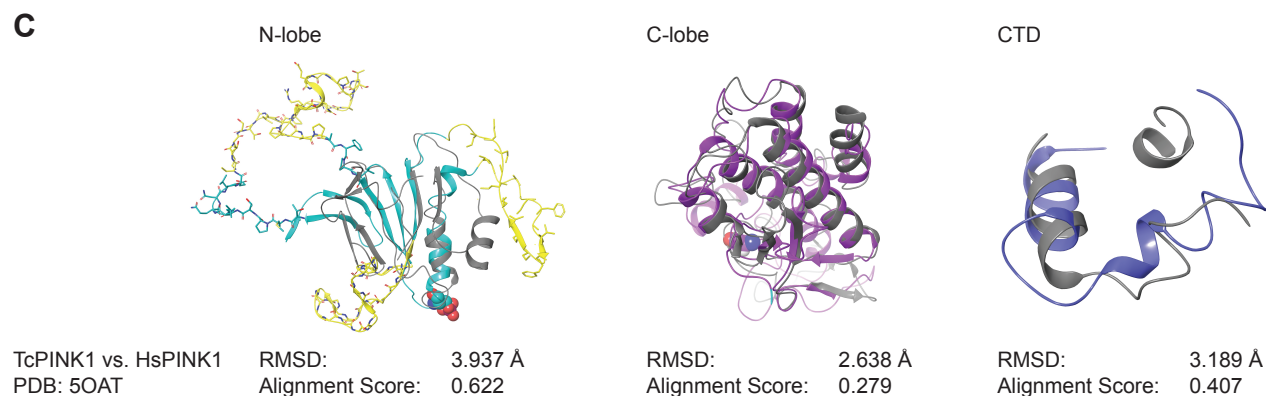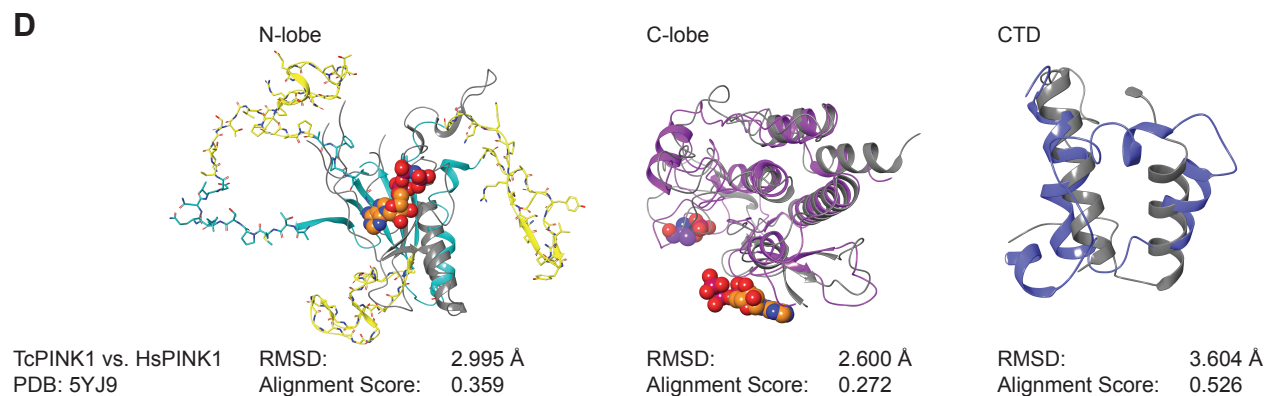

Supplement: Supplemental Material [file KAUP_A_2151294_SM8220.zip › PINK1Gly411_FigureS4.pdf]

SUPPLEMENTARY FIGURE S3

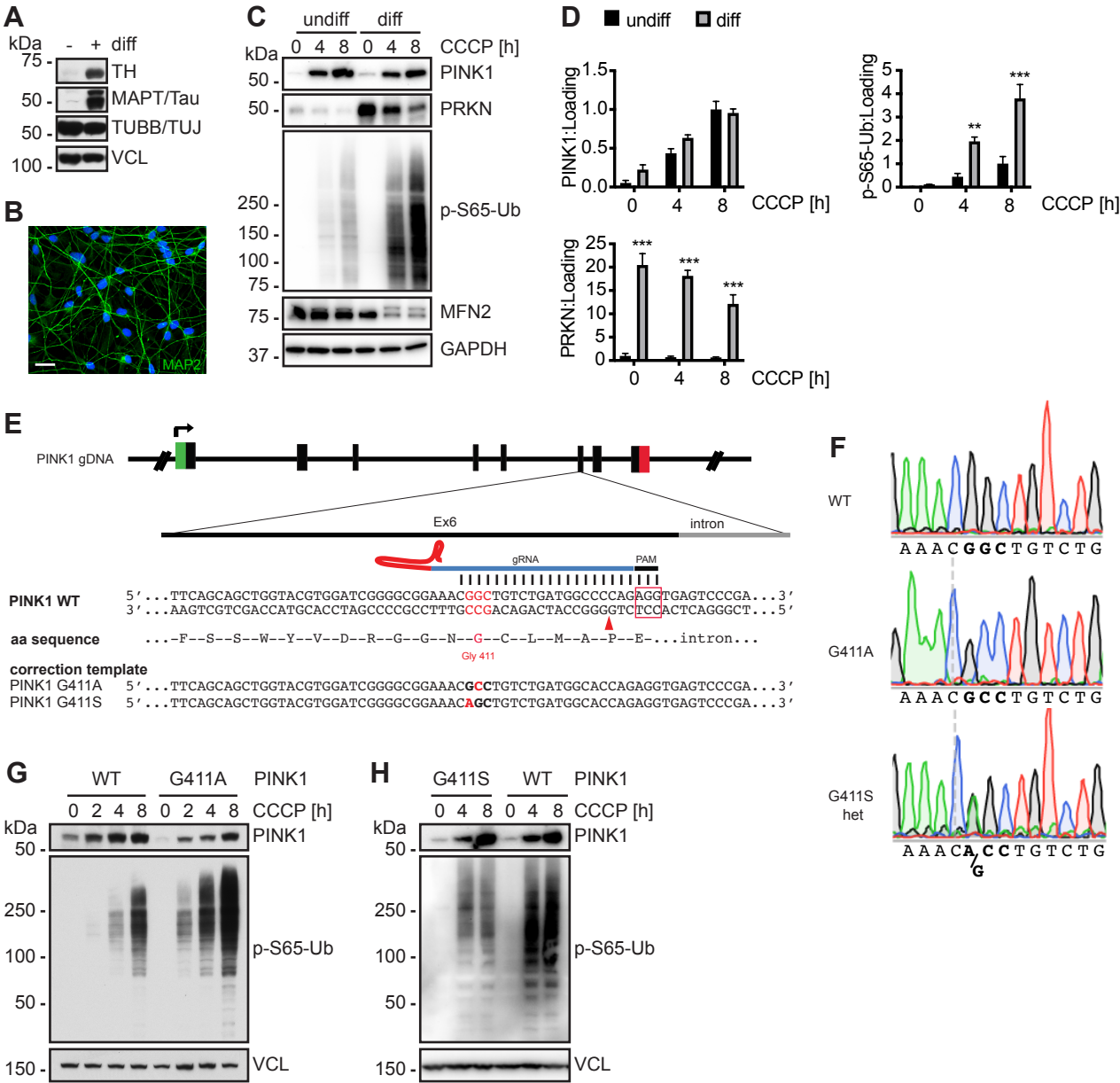

Supplement: Supplemental Material [file KAUP_A_2151294_SM8220.zip › PINK1Gly411_FigureS3.pdf]

SUPPLEMENTARY FIGURE S2

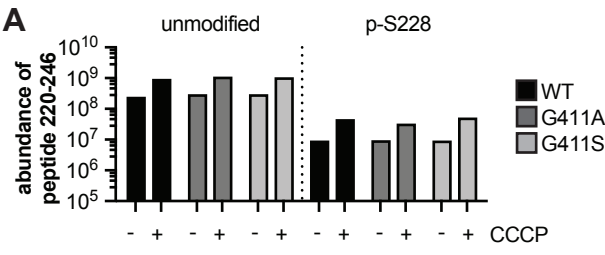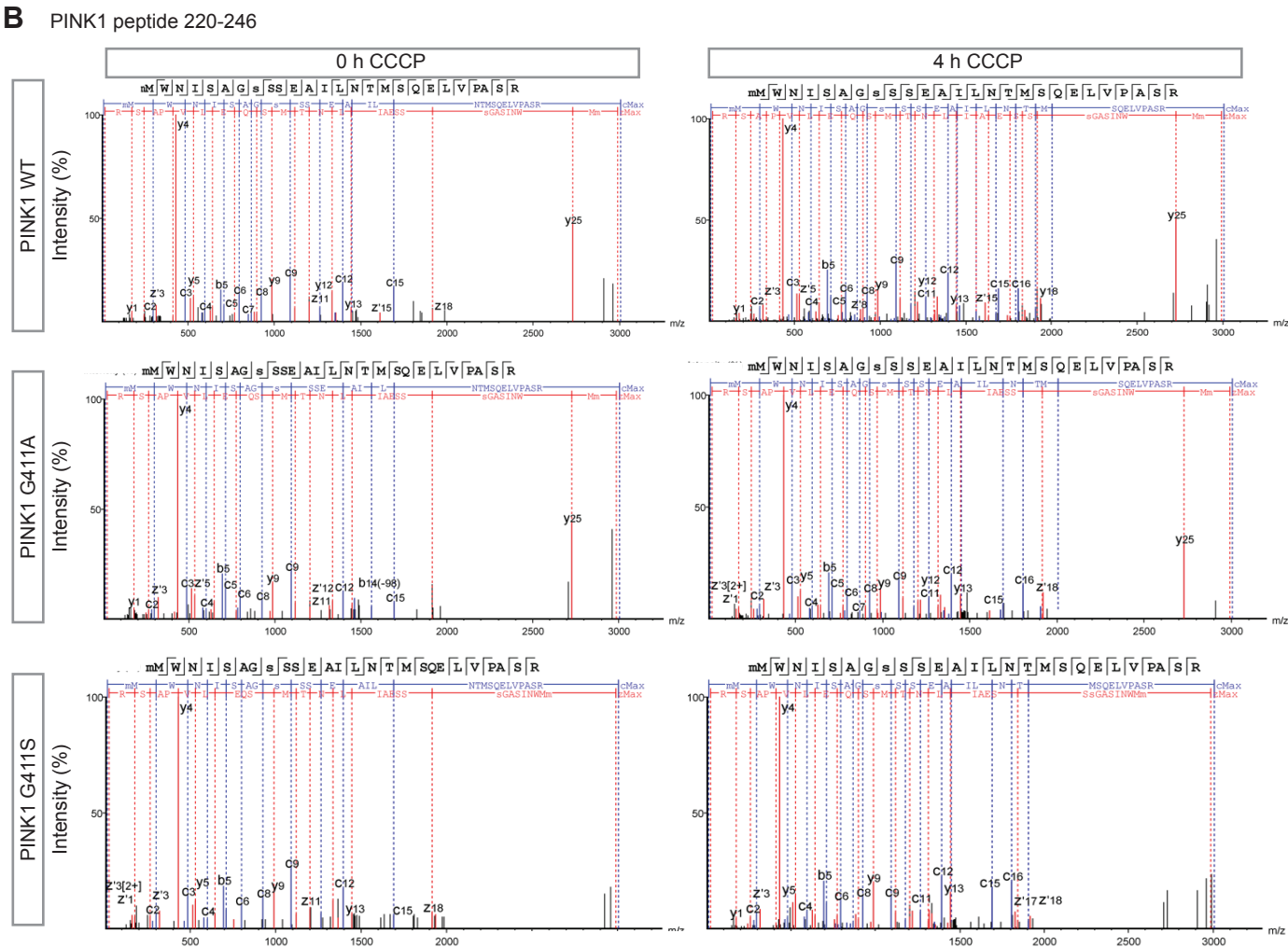

Supplement: Supplemental Material [file KAUP_A_2151294_SM8220.zip › PINK1Gly411_FigureS2.pdf]

# SUPPLEMENTARY FIGURE S1

**A**

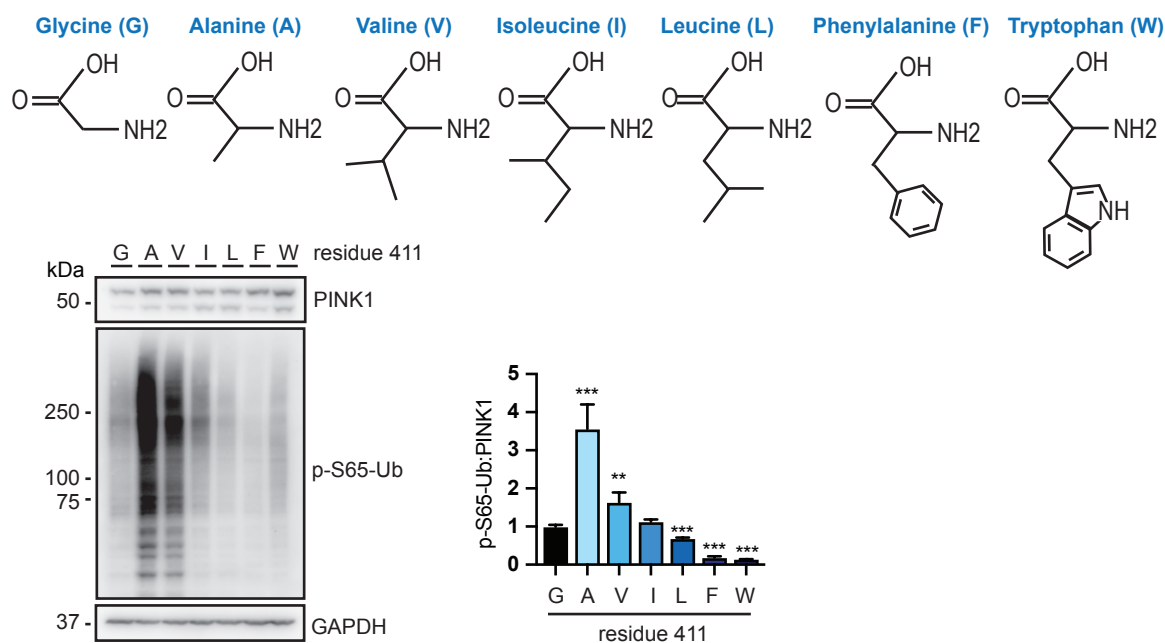

**B**

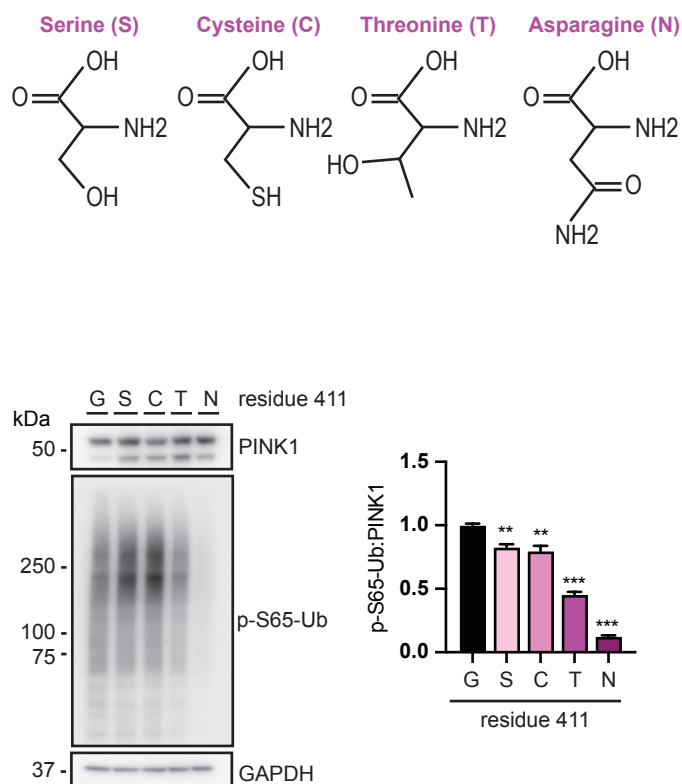

**C**

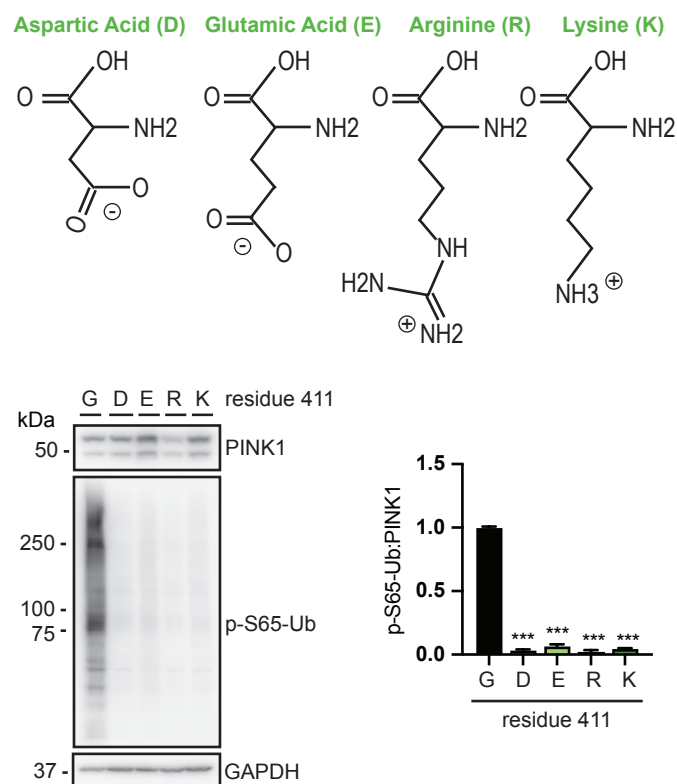

Supplement: Supplemental Material [file KAUP_A_2151294_SM8220.zip › PINK1Gly411_FigureS1.pdf]
